# Resolving tumor microenvironment heterogeneity to forecast immunotherapy response in triple-negative breast cancer through multi-scale analysis

**DOI:** 10.3389/fonc.2025.1538574

**Published:** 2025-08-19

**Authors:** Shihao Sun, Shuang Chen, Kaiyuan Li, Ge Zhang, Nan Wang, Yijia Xu, Xinxing Wang, Jiangrui Chi, Lin Li, Yi Sun

**Affiliations:** ^1^ Department of Breast Surgery, The First Affiliated Hospital of Zhengzhou University, Zhengzhou, Henan, China; ^2^ Center of Reproductive Medicine, The First Affiliated Hospital of Zhengzhou University, Zhengzhou, Henan, China; ^3^ Department of Thoracic Surgery, The First Affiliated Hospital of Zhengzhou University, Zhengzhou, Henan, China; ^4^ Department of Cardiology, Henan Province Clinical Research Center for Cardiovascular Diseases, Zhengzhou, China; ^5^ Department of Cardiology, The First Affiliated Hospital of Zhengzhou University, Zhengzhou, China; ^6^ Department of Cardiology, Key Laboratory of Cardiac Injury and Repair of Henan Province, Zhengzhou, China

**Keywords:** triple-negative breast cancer, BRCA1 mutation, tumor microenvironment, cancer-associated fibroblasts, immune checkpoint inhibitor

## Abstract

**Background:**

Immunotherapy has been used in the clinical management of TNBC. While BRCA1 mutations are associated with immunotherapy response, the therapeutic outcomes in TNBC patients are not promising.

**Methods:**

This study integrated spatial, single-cell, and bulk RNA-seq data to explore the role of BRCA1 in reshaping the TNBC microenvironment. Through multi-scale analysis, phenotype changes and potential biomarkers in cancer-associated fibroblasts (CAF) were identified. To validate these findings at the protein level, we employed high-resolution, label-free proteomics sequencing in our in-house cohort, providing critical real-world validation. A predictive system for response to ICIs was constructed through the step-by-step machine learning pipeline.

**Results:**

Compared to BRCA1 mutant patients, BRCA1 wild-type patients experienced increased T-cell exhaustion and dendritic cell tolerance. We identified a MEG3+ pre-CAF subgroup via pseudo-time analysis. Moreover, ISG15 may serve as an immunoregulatory biomarker, and the proposed predictive model demonstrated potential in forecasting immunotherapy response, although further validation is needed.

**Conclusions:**

This study highlighted the cellular heterogeneity of TNBC and identified ISG15 as a candidate biomarker potentially associated with treatment response. The ISG15-based predictive system might provide a robust framework for predicting ICI response.

## Introduction

Breast cancer remains the most frequently diagnosed malignancy and the leading cause of cancer-related mortality among women worldwide (1). Among its molecular subtypes, triple-negative breast cancer (TNBC), defined by the absence of estrogen receptor (ER), progesterone receptor (PR), and human epidermal growth factor receptor 2 (HER2), exhibits pronounced heterogeneity and aggressive clinical behavior. Compared with luminal A, luminal B, and HER2-positive subtypes, TNBC is associated with significantly poorer outcomes (2–4). The 5-year survival rate for TNBC is approximately 77%, which is 8% to 16% lower than that of hormone receptor–positive breast cancers (5, 6). TNBC also demonstrates markedly higher risks of early relapse and distant metastasis (5). Approximately 25% of patients with TNBC experience disease recurrence (4), compared to a recurrence rate of ~15% across all breast cancer types (7). In terms of locoregional recurrence, TNBC and HER2-positive subtypes show substantially elevated rates (7.6% and 7.5%, respectively), whereas luminal A and B subtypes have considerably lower rates (1.5% and 2.9%, respectively) (8). Moreover, nearly 40% of patients with stage I–III TNBC relapse within 2 to 3 years after receiving standard therapy (9). These clinical patterns underscore an imperative need for more effective therapeutic strategies in TNBC, with immunotherapy emerging as a particularly promising option.

In recent years, immune checkpoint inhibitors (ICIs) have revolutionized cancer therapy, particularly for solid tumors with high mutational loads, representing a major breakthrough in oncology. TNBC is notably suited for this therapy due to its high tumor mutational burden, increased tumor-infiltrating lymphocytes, and enhanced PD-L1 expression, all of which heighten its immunogenicity (10–12).

Approximately 10-20% of TNBC tumors are characterized by BRCA1 deficiency due to epigenetic modification or mutation (13). These mutations impair homologous recombination repair, further increasing sensitivity to DNA-damaging agents including platinum drugs and PARP inhibitors (14). BRCA1-mutated TNBC exhibits a unique tumor immune microenvironment characterized by higher mutational loads and extensive immune lymphocyte infiltration, suggesting that BRCA1 mutations could serve as potential biomarkers for ICI responses. Recently, an *in vivo* study demonstrated that treatment with ICIs and platinum-based chemotherapy significantly reduces tumor growth and improves survival rates in BRCA1-deficient TNBC mice (15). However, a comprehensive view of the tumor microenvironment (TME) and the interplay of tumor, immune, and stromal cells of BRCA1 mutated tumors have not yet been described.

This study used scRNA-seq data from TNBC patients with BRCA1 mutations (BRCA1-MT) or wild-type (BRCA1-WT) to explore TME heterogeneity and cellular interactions. Multi-scale analysis was performed to explore the difference of cancer-associated fibroblasts (CAFs) in two groups and further identified ISG15 as a key driver biomarker. Finally, we developed a predictive system for response to immunotherapy via our proposed machine learning pipeline, aiming to precisely identify individuals who benefit from ICI therapy.

## Materials and methods

### Data acquisition and processing

We extracted and analyzed the sc-RNAseq data involved in this study from the Gene Expression Omnibus (GEO) database under the accession number GSE161529, encompassing four samples with BRCA1-mutant (BRCA1-MT1-4) and four samples with BRCA1 wild (BRCA1-WT1-4). Seurat package was used to generate the Seurat objects containing scRNA-seq gene expression matrices for main cell types. Low-quality cells were removed from each sample according to nFeature, nUMI per cell and, mitochondria content (**Supplementary Figure S1**). Further, the expression matrices underwent normalization and scaling via the NormalizeData and ScaleData functions in the Seurat package. According to the top 2000 highly variable genes and 30 principal components, reduce dimension was employed via the Uniform Manifold Approximation and Projection (UMAP) algorithm. Harmony algorithm (16) was conducted to counteract the batch effect. The marker genes for each cell cluster were identified through the FindAllmarkers function, with the cutoff criteria as log_2_FC > 0.25 and false discovery rate (FDR) < 0.05. With the aid of well-known cell markers and singleR package (17), major cell populations were annotated. The clustering of each major cell type was subsequently re-clustered using the workflow described above. These new clusters were identified as “subclusters”, representing different phenotypes within each major cell type. Tissue enrichment was assessed using the ratio of observed to expected cell number (Ro/e) in each tissue type. Specifically, Ro/e > 1 indicates enrichment of the cell cluster in that tissue, whereas Ro/e < 1 suggests under-representation (18).

The filtered spatial transcriptome data was fetched from the website, https://zenodo.org/record/4739739/. Seurat package was used to conduct subsequent analysis. According to the top 3000 highly variable genes, the expression matrix of each slice was normalized and scaled utilizing the SCTransform function. Subsequently, PCA dimensionality reduction analysis was performed for building an SNN graph with the default parameter. Using the anchor-based integration pipeline within Seurat package, we established a mapping between the spatial RNA-seq and the scRNA-seq data, yielding the prediction scores of subcluster to every spot within each slice.

### Function enrichment analysis

Gene ontology (GO) analysis was conducted via clusterProfiler package (19). We utilized gene set variation analysis (GSVA) to calculate the enrichment scores of hallmark pathways for each cell. The differential activating pathways between two cell subclusters were identified using the limma package (20). The significant terms and pathways were identified with the cutoff of adjusted p-value <0.05.

### Trajectory analysis of cell differentiation

The R packages, monocle2, and monocle3 were applied to explore the trajectory of cell differentiation among the selected clusters (21). For monocle2, the DDRtree algorithm was employed to reduce the dimensions. The mutual nearest neighbor algorithm was used to eliminate the batch effect for monocle3.

### Differential abundance analysis of cell neighborhoods via milo

We utilized the milo algorithm for identifying the differential abundance of cell neighborhoods in two distinct conditions (22). More specifically, a KNN graph based on scRNA-seq data was constructed, followed by the data split into cell neighborhoods and the differential abundance analysis of selected cell subclusters was performed. In this section, we configured the parameters with k=40 and d=30, following the author’s recommendation.

### Identification of malignant cells

The copykat software was utilized to infer genome-wide copy number variations from the single-cell gene expression count matrix (23). Immune cells were employed as reference cells, where single aneuploid cells with copy number variations were deemed as tumor cells, and diploid cells were predicted as normal epithelial cells. The CytoTRACE (24) algorithm was employed to validate the tumor cell predictions derived from CopyKAT algorithm.

### Gene regulatory network analysis

For quantifying the difference in transcription factors (TF) activities between distinct cell clusters, cluster-specific gene regulatory networks were built utilizing pySCENIC (25) package with default parameters. Count matrix of scRNA-seq data was extracted from Seurat object and converted to the Loom format file as input for downstream analysis. In particular, we first utilized GRNBoost2 to form a co-expression network, followed by discovering regulons for every TF via RcisTarget. The motif database of humans is accessible through the website https://resources.aertslab.org/cistarget/databases/homo_sapiens/hg38/. The activity of each regulon was measured through the AUCell algorithm. Subsequently, binary values for each regulon were obtained using AUC score thresholds automatically determined in the process. For one specific subcluster, we regarded TFs that activated in over 20% of cells in at least one subcluster as significant.

### Detection of intratumoral and intertumoral heterogeneity

To explore the distinct levels of tumor heterogeneity between the BRCA1-WT group and the BRCA1-MT group, we calculated the intratumoral and intertumoral heterogeneity scores as previously reported (26). Notably, malignant tumor cells based-CopyKAT were chosen and re-clustered using the method described above with default parameters. Extracting the top 50 principal components as features for calculating the heterogeneity score. To ensure consistency in data scaling, z-score standardization is used for all heterogeneity scores.

### High-dimensional weighted gene co-expression network analysis

The hdWGCNA package (27), which was designed for analyzing high-dimensional scRNA-seq data, was deployed to construct a scale-free network at the single-cell level utilizing default parameters.

### Multi-scale identification framework

In our effort to identify the critical molecular marker in one cell subgroup, we developed a reliable analysis pipeline via employing multi-scale data, encompassing bulk and scRNA-seq data. Initially, we identified the marker genes of myCAF utilizing FindAllMarkers function, with the threshold as adjusted p-value<0.05 and log2FC>0.25. Following this, Differential module eigengene analysis was conducted to discover the myCAF and BRCA1-WT specific module. For each module, the top 200 hub genes were extracted. Upon the overlap genes of these myCAF marker genes and hub genes (**Supplementary Table S1**), the differential expression analysis was employed between BRCA1-WT samples and BRCA1-MT samples within the METABRIC dataset. The substantially elevated genes in the BRCA1-WT group were regarded as the key genes (**Supplementary Table S2**).

### Reconstruction of the ICI response predictor

Thirteen ICI pre-therapy RNA-seq cohorts with response information (n = 829) were collected in this study (**Supplementary Table S3**). The expression values of RNA-seq data for each cohort were converted to TPM values, followed by log2-transformed. To eliminate unnecessary interference, we utilized the Combat function in the sva package to integrate the four cohorts encompassing Braun 2020 (n = 172) (28), Mariathasan 2018 (n = 298) (29), Liu 2019 (n = 119) (30), and Pender 2021 (n = 72) (31). We randomly allocated the integrated cohort (n = 661) into an 80% training set and a 20% validation set. Model performance was streamlined via 5-fold cross-validation. Utilizing the tidymodels package, eight machine learning algorithms were implemented to derive the predictive models for ICI therapy response. These algorithms included Support Vector Machine (SVM), Extreme Gradient Boosting (XGBoost), Random Forest (RF), LightGBM, naive Bayes (NB), multilayer perceptron (MLP) neural network, K-Nearest Neighbour (KNN). In terms of the validation set, the model with the maximum receiver operating characteristic curve (ROC) was deemed as optimal.

### High-sensitivity label-free quantitative proteomics analysis

After surgically proving, paired tumor (n=9) and para-carcinoma tissues (n=9) from breast cancer patients were collected from the First Affiliated Hospital of Zhengzhou University. Samples were obtained with informed consent. This study was approved by the Ethics Committee of the First Affiliated Hospital of Zhengzhou University (Ethics review number: 2024-KY-0549; Zhengzhou, China). All methods were conducted in accordance with the relevant guidelines and regulations. Subsequently, the protein expression was quantified via high-sensitivity label-free quantitative proteomics sequencing, as described in the **Supplementary Methods**.

### Statistical analysis

Fisher’s exact test was implemented for categorical variables, whereas Student’s t-test, Wilcoxon rank-sum test, ANOVA test, and Kruskal-Wallis test were conducted for continuous variables. Spearman correlation was used for continuous versus continuous variables. Survival analysis was performed through Kaplan-Meier curves and log-rank tests. To correct for multiple tests, the p-values were adjusted to the FDR utilizing the Benjamini-Hochberg approach where appropriate. Unless otherwise indicated, all statistical tests were two-tailed. Significance levels are indicated by asterisks (*p <0.05; **p <0.01; ***p <0.001, ****p <0.0001). Statistical and bioinformatics analyses mentioned above were carried out with R software (version 4.2.1).

## Results

### The single-cell atlas of BRCA1 MT and BRCA1 WT samples

**Figure 1:**
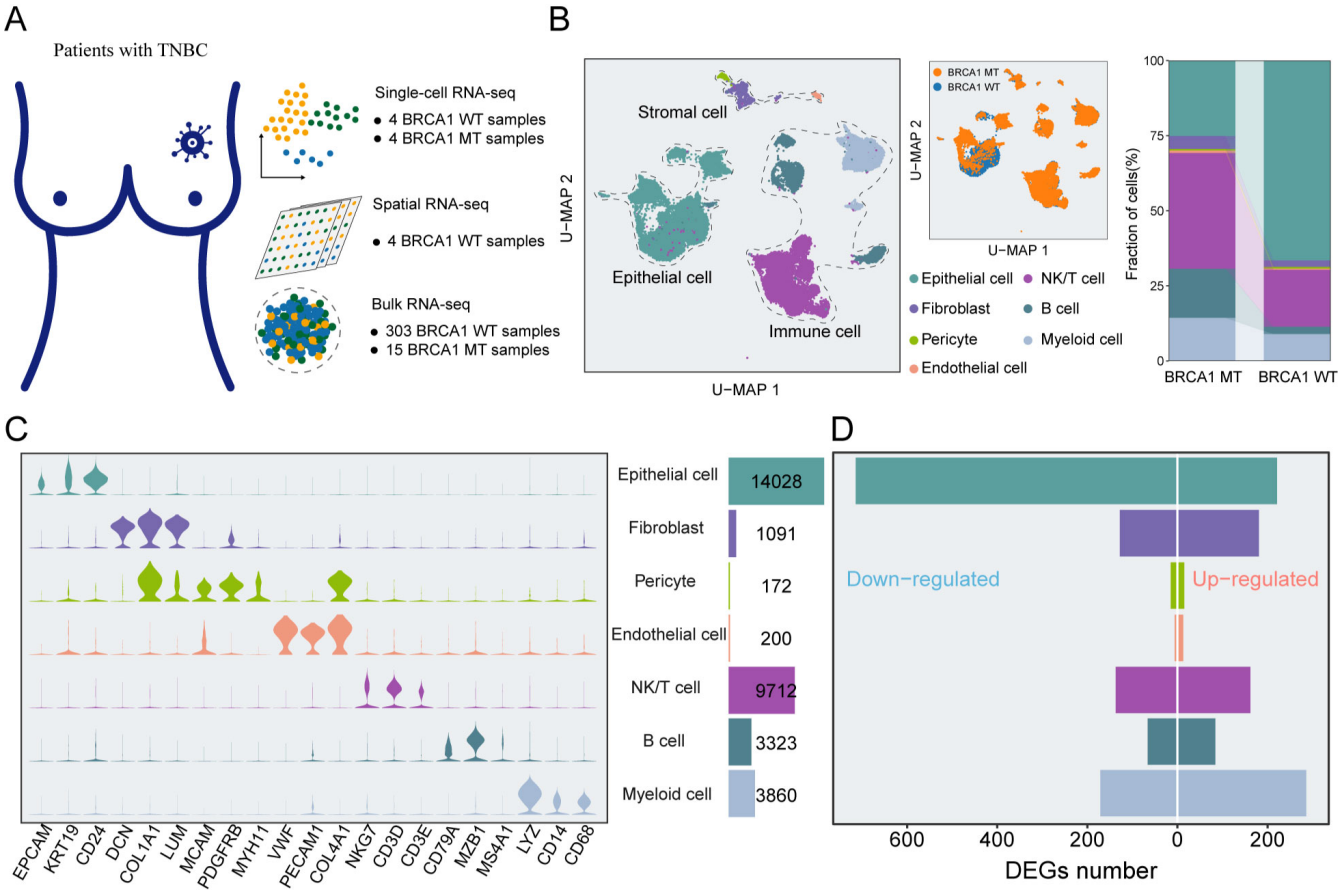
An atlas of cellular heterogeneity from BRCA1-WT and BRCA1-MT patients. **(A)** The acquisition of multi-scale transcriptomic datasets from TNBC specimens. **(B)** UMAP plot of the 32,386 cells from eight TNBC samples, indicating seven main clusters (left panel). The proportions of distinct cell clusters (right panel). Different colors indicate cell clusters and BRCA1 mutation status. **(C)** Feature plots of canonical marker genes for every cell cluster. **(D)** Bar plots of the DEGs in each cell cluster.

To understand gene-expression perturbations and generate a comprehensive map of the TME landscape of TNBC, we collected and analyzed eight specimens, comprising four samples with BRCA1-mutant (BRCA1-MT1-4) and four samples with BRCA1 wild (BRCA1-WT1-4) (**Figure 1A**). After filtering low-quality cells through a rigorous quality control pipeline, 32,386 cells remained for downstream analysis (**Supplementary Figures S1A, B**). Of these, 18,182 cells were derived from BRCA1-MT samples, while 14,204 cells were obtained from BRCA1-WT samples. The mean number of genes examined per cell was 1,331. Afterward, the standard workflow in Seurat was utilized to identify the subpopulations within scRNA-seq data, yielding seven unique cell subsets (**Figure 1B**). Leveraging the expression of well-characterized markers, we identified cell lineages, including Epithelial cells, immune cells (NK/T cells, B cells, and Myeloid cells), stromal cells (Fibroblasts, pericytes, and endothelial cells) (**Figure 1C**, **Supplementary Figure S2**).

To gain further insight, we assess the difference in TME landscape between the BRCA1-WT group and the BRCA1-MT group, and the results indicated that the significantly elevated percent of immune and stromal cells were observed in the BRCA1-MT group, comparing to BRCA1-WT group with more epithelial cells (**Figure 1B**). Next, differential expression analysis for each cluster was executed to decode the transcriptional perturbations caused by BRCA1 mutation, totally yielding 2,199 differentially expressed genes (DEGs) (**Figure 1D**). Regarding epithelial cells, BRCA1-WT samples harbored more up-regulated DEGs than BRCA1-MT samples. Of note, upregulated genes in BRCA1-WT were mainly enriched in immune cells and Fibroblasts. Although BRCA1-WT tumors showed a higher proportion of epithelial cells, the functional disturbances reflected by DEGs were predominantly associated with immune and stromal compartments. This may suggest a compensatory or stress-induced transcriptional response in these less abundant cell types, highlighting the importance of integrating both compositional and functional perspectives. Altogether, our findings dedicated the cellular heterogeneity within TME caused by BRCA1 mutation, which was meticulously analyzed in the subsequent section.

### T cells in BRCA1-WT specimens exhibit a more exhausted and dysfunctional phenotype

**Figure 2:**
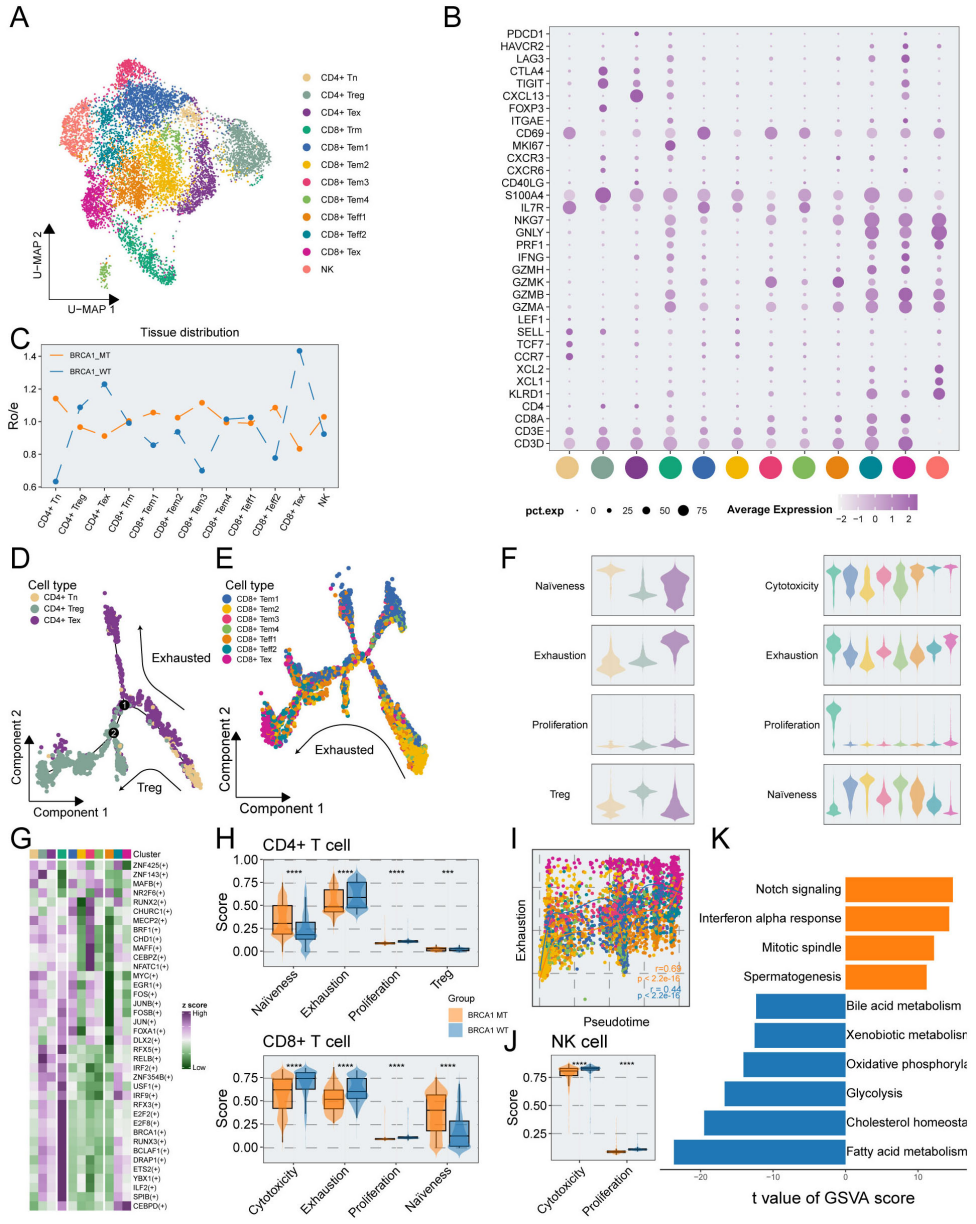
Phenotypic and functional characterization of T/NK cells in BRCA1-WT and BRCA1-MT samples. **(A)** UMAP plot showing the distribution of T/NK cells, colored by cell subcluster. **(B)** Average gene expression of selected marker genes for T/NK cell subclusters. **(C)** The tissue prevalence for each cell subcluster estimated via Ro/e analysis. **(D)** The developmental trajectory of CD4+ T cells, colored by cell subclusters from the associated cell subpopulations. **(E)** The developmental trajectory analysis of CD8+ Tem, Tex, and Teff cells, colored by cell subclusters from the associated cell subpopulations. **(F)** Violin plot showing the signature scores of gene sets associated with naiveness, cytotoxicity, exhaustion, proliferation, and Treg in each T cell subpopulations, colored by cell subpopulations in **(A)**. **(G)** Heatmap showing the differentially activated TFs in each T cell subpopulations. **(H)** Signature scores of CD4+ cells (top panel), and CD8+ T cells (bottom panel) compared between the BRCA1-WT and BRCA1-MT groups. Wilcoxon rank-sum test. **(I)** Scatter plot showing the correlation of pseudotime and exhaustion score of CD8+ Tem, Tex, and Teff cells, colored by cell subpopulations in **(A)**. **(J)** Signature scores of NK cells compared between the BRCA1-WT and BRCA1-MT groups. Wilcoxon rank-sum test. **(K)** Bar plot showing the differentially activated pathways compared between BRCA1-WT and BRCA1-MT groups, colored by groups in **(H)**. *** p<0.001, **** p<0.0001.

To investigate the relative heterogeneity of immune cells between BRCA1-WT and BRCA1-MT samples, we explored the features and functions of T/NK cells. T/NK cells were re-clustered into 12 subpopulations, encompassing CD4+ naïve T cells (CD4+ Tn), CD4+ exhausted T cells (CD4+ Tex), CD4+ regulatory T cells (CD4+ Treg), CD8+ resident memory T cells (CD8+ Trm), four clusters of CD8+ effector memory T cell (CD8+ Tem), two clusters of CD8+ effector T cells (CD8+ Teff), CD8+ exhausted T cells (CD8+ Tex), NK cells, which displayed characteristic marker gene expression (**Figures 2A, B**, **Supplementary Figure S3A**). Notably, CD4^+^ Treg, CD4^+^ Tex, and CD8^+^ Tex cells exhibited high expression of canonical exhaustion markers, including PDCD1, HAVCR2, LAG3, CTLA4, and TIGIT (**Figure 2B**). Ro/e analysis further revealed that these subpopulations were preferentially enriched in the BRCA1-WT group, indicative of a more exhausted phenotype (**Figure 2C**). Furthermore, we found that the proportion of CD4+ Tex cells was positively correlated with the proportion of CD4+ Treg cells (**Supplementary Figure S3B**). The pseudotime analysis was conducted to deeply understand the immune dynamics. The results revealed that there were potential developmental branches, from CD4+ Tn to CD4+ Treg (Path1) or CD4+ Tex (Path2) (**Figure 2D**). We also identified the differential expression levels of marker genes during the differentiation and showed that FOXP3 and ICOS were strikingly upregulated along the branch of CD4+ Treg, consistent with their established roles in defining and sustaining the Treg lineage (**Supplementary Figure S3C**). It was suggested via trajectory analysis that the developmental trajectories were unclear in CD8+ T cells (**Supplementary Figure S3D**). Moreover, we employed a trajectory analysis based on CD8+ Tem and Teff cells, and the findings illustrated an apparent tendency to transform into CD8+ Tex subpopulation (**Figures 2E, F**). Moreover, Slingshot-based trajectory analysis was applied to further corroborate these results (**Supplementary Figures S3E, F**). Hence, we hypothesized that CD8+ Tex cells might derive from CD8+ Tem cells in TNBC, simultaneously developing the property of elevated exhaustion.

To assess the functional phenotype of T cells, we measured the T-cell-associated signature scores via ssGSEA. The results delineated that both CD4+ Tex and CD8+ Trm subpopulations had higher proliferation scores. The highest cytotoxicity scores were enriched in CD8+ Teff subpopulation, showing the key cell killing capability (**Figure 2F**). For CD8+ T cells, we found that the cytotoxicity score was negatively associated with naïveness score, in line with the poor cell killing capability of more naïve T cells (**Supplementary Figure S3G**). It was revealed via GO analysis of CD8+ T cells that pathways linked to immune negative regulation were preferentially congregated in Tex cells, metabolism-associated pathways were predominantly observed in Trm cells, and T cell activation pathways were enriched in other subpopulations (**Supplementary Figure S3H**). Signature scores were compared between the BRCA1-WT and BRCA1-MT groups. As depicted in **Figure 2H**, the naïve scores of T cells were substantially elevated in the BRCA1-MT group compared to the BRCA1-WT group, while higher exhaustion and proliferation scores of T cells were observed in the BRCA1-WT group. Intriguingly, we found that the cytotoxicity score of CD8+ T cells in the BRCA1-WT group was significantly higher than the BRCA1-MT group (**Figure 2H**). Consistent with prior research, dysfunctional T cells displayed higher proliferative potential (**Figures S3I, J**).

For Tem, Teff, and Tex subpopulations of CD8+ T cells, we dug into the dynamic landscape of exhaustion scores along developmental trajectory via pseudotime analysis, discovering a significantly more rapid ascent of exhaustion scores in BRCA1-WT group (**Figure 2I**). SCENIC analysis was conducted to further unravel the underlying mechanism of a more dysfunctional phenotype in the BRCA1-WT group. Specifically, genes within the interferon regulatory factor family (including IRF2 and IRF9) exhibited increased transcriptional activity in dysfunctional T cells which preferentially enriched in BRCA1-WT group (**Figure 2G**). Notably, this activation pattern was also confirmed at the gene expression level. We compared the expression profiles of IRF family genes in exhausted T cells between the two groups, and the results were consistent with our hypothesis (**Supplementary Figure S4**), collectively contributing to the formation of the immune-suppress microenvironment (32, 33). These findings illustrated that BRCA1 potentially induced the immune dysfunction of T cells, leading to a better understanding for the immunotherapy of BRCA1-associated TNBC.

In addition, we delved into the underlying function of NK cell subpopulation between the two groups. It was revealed by GSVA that the BRCA1-WT group was characterized by more activated metabolism-related pathways, such as fatty acid metabolism, glycolysis, and oxidative phosphorylation (**Figure 2K**). This result was supported by the finding of elevated cytotoxicity scores of NK cells in the BRCA1-WT group (**Figure 2J**). Taken together, these evidences indicated the activation of NK cells in the BRCA1-WT group.

### Activated phenotype of TAMs in the BRCA1-MT group and immune tolerogenic phenotype of DCs in the BRCA1-WT group

**Figure 3:**
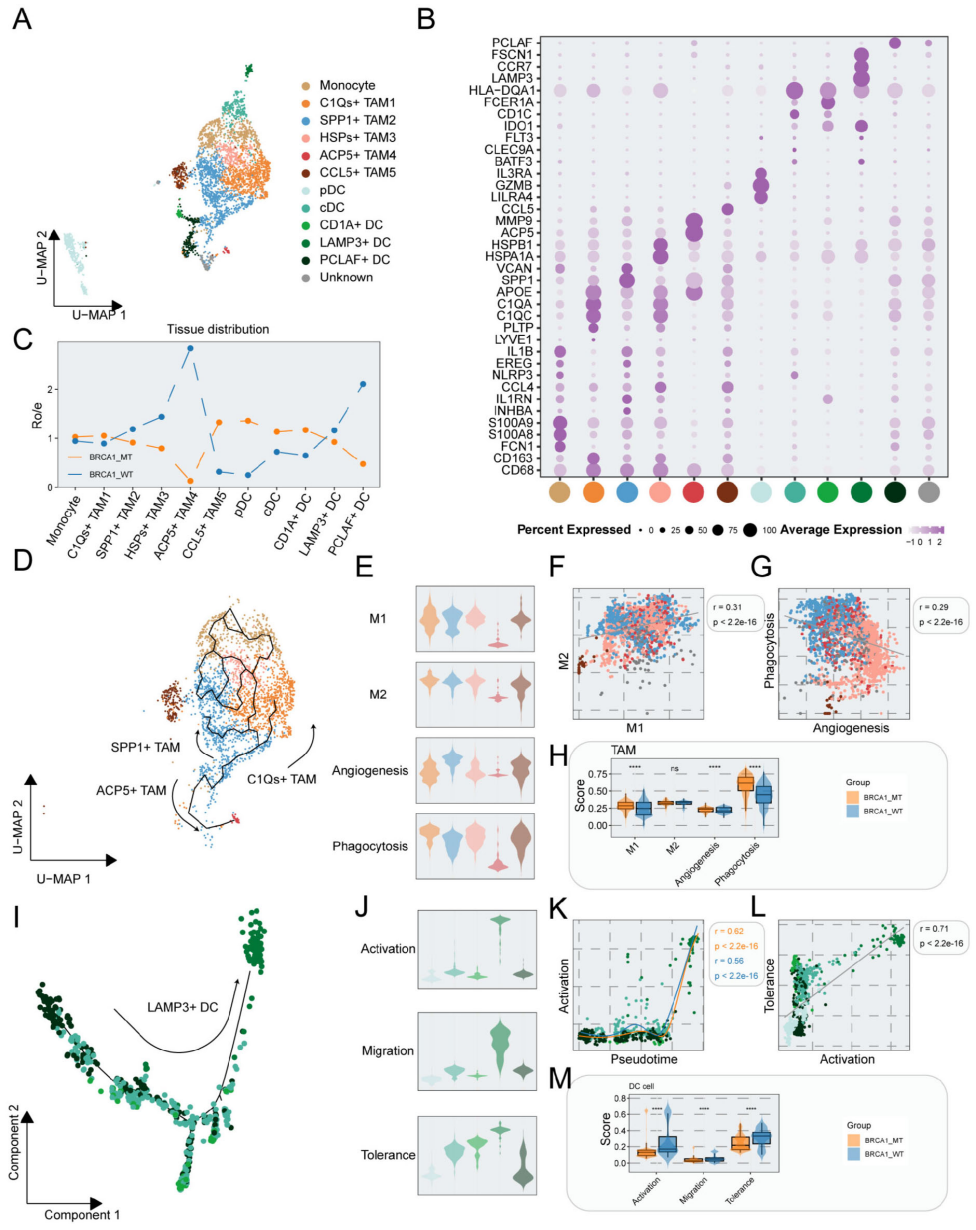
The characterization of myeloid cells in BRCA1-WT and BRCA1-MT samples. **(A)** UMAP plot showing the distribution of myeloid cells, colored by cell subpopulations. **(B)** Average gene expression of selected marker genes for myeloid subclusters. **(C)** The tissue prevalence for each cell subcluster was calculated via Ro/e analysis. **(D)** The developmental trajectory of monocytes, and TAMs, colored by cell subpopulations in **(A)**. **(E)** Violin plot showing the signature scores of gene sets associated with M1, M2, angiogenesis, and phagocytosis in each TAMs cell subpopulations, colored by cell subpopulations in **(A)**. **(F)** Scatter plot showing the correlation of M1 and M2 signature scores in TAMs, colored by cell subpopulations in **(A)**. **(G)** Scatter plot showing the correlation of angiogenesis and phagocytosis signature scores in TAMs, colored by cell subpopulations in **(A)**. **(H)** Signature scores of TAMs were compared between the BRCA1-WT and BRCA1-MT groups. Wilcoxon rank-sum test. **(I)** The developmental trajectory of DCs, colored by cell subpopulations in **(A)**. **(J)** Violin plot showing the signature scores of gene sets associated with activation, migration, and tolerance in each DC cell subpopulation, colored by cell populations in **(A)**. **(K)** Scatter plot showing the correlation of pseudotime and activation score in DCs, colored by cell subpopulations in **(A)**. **(L)** Scatter plot showing the correlation of activation and tolerance signature scores in DCs, colored by cell subpopulations in **(A)**. **(M)** Signature scores of DCs compared between the BRCA1-WT and BRCA1-MT groups. Wilcoxon rank-sum test. **** p<0.0001.

Accumulating evidences indicated that myeloid cells are the most abundant leukocytes in breast tumours (34), and we herein decoded the cellular complexity of myeloid cells in the patients with BRCA1-WT and BRCA1-MT. Myeloid cells were subclustered into monocyte, Tumor-associated macrophage (TAM) including SPP1+ TAM, C1Qs+TAM, HSPs+ TAM, ACP5+ TAM, and CCL5+ TAM, classical dendritic cell (cDC) encompassing cDC, CD1A+ DC, LAMP3+ DC and PCLAF+ DC, plasmacytoid DC (pDC) as well as unknown subpopulations (**Figure 3A**). Combined with the marker genes of each subpopulation (**Figure 3B**, **Supplementary Figure S5A**), the unknown cell subpopulation was regarded as double cells and excluded from the subsequent analysis.

In contrast to enhanced transcriptional perturbations of myeloid cells within the BRCA1-MT group (**Figure 1D**), the Ro/e analysis demonstrated that TAMs predominantly enriched in the BRCA1-WT group but not C1Qs+ TAM and CCL5+ TAM (**Figure 3C**). The trajectory analysis of monocytes and TAMs indicated that along seperate developmental paths, monocytes derived from three TAM subpopulations including SPP1+ TAM, C1Qs+ TAM, and ACP5+ TAM (**Figure 3D**). Subsequently, the signature scores in each TAM subpopulation were evaluated to illustrate the functional phenotype. As depicted in **Figure 3E**, the M1 and M2 scores showed no significance among these TAM subpopulations. However, we found a strikingly positive correlation between M1 and M2 scores, reflecting the cellular heterogeneity of TAMs within TME (r=0.31, p < 0.001) (**Figure 3F**). In response to the function of their respective marker genes, we observed that SPP1+ TAMs showed increased angiogenesis scores, whereas C1Qs+, HSPs+, and CCL5+ TAM subpopulations showed increased phagocytosis scores (**Figure 3E**). In contrast with the relationship between M1 and M2 scores, a significant negative correlation was observed in phagocytosis and angiogenesis scores, indicating their superiority in illustrating the features of TAMs (r = -0.29, p < 0.001) (**Figure 3G**). BRCA1 mutation might comprehensively enhance the activation of TAMs within the TME, as supported in **Figure 3H**. TAMs from BRCA1-MT tumors exhibited significantly higher M1 polarization scores, as well as elevated angiogenesis and phagocytosis scores, compared to those from BRCA1-WT tumors (**Figure 3H**). These results indicate that TAMs in the BRCA1-MT group are functionally more active, potentially contributing to the dynamic remodeling of the immune landscape.

We then interrogated the features of DC subpopulations and conducted a comparison between the two groups. Monocle3 discovered that PCLAF+ DC cells with high proliferation differentiated into LAMP3+ DC cells with comprehensively elevated function along pseudotime trajectory, which was validated by correlation analysis between signature scores and pseudotime (**Figures 3I-K**, **Supplementary Figures S5B**-**S4C**). Besides, there was a significant positive link between activation score and migration score as well as the tolerance score of DC cells (**Figure 3L**, **Supplementary Figure S5D**). PCLAF+ DC, as an immature DC subpopulation enriched in the BRCA1-WT group, induced the proliferation of Treg cells through interacting with Tumor cells (**Figure 3C**) (35). Awing to the immature property of PCLAF+ DC, we excluded this subpopulation and conducted the comparison of signature scores between the two groups. The result indicated that all three signature scores were strikingly higher in the BRCA1-WT group than in the BRCA1-MT group (**Figure 3M**). Taken together, we proposed the hypothesis that activated DCs in the BRCA1-WT group could mediate immune tolerance within the TME.

### A subgroup of novel B cells in the BRCA1-WT group

**Figure 4:**
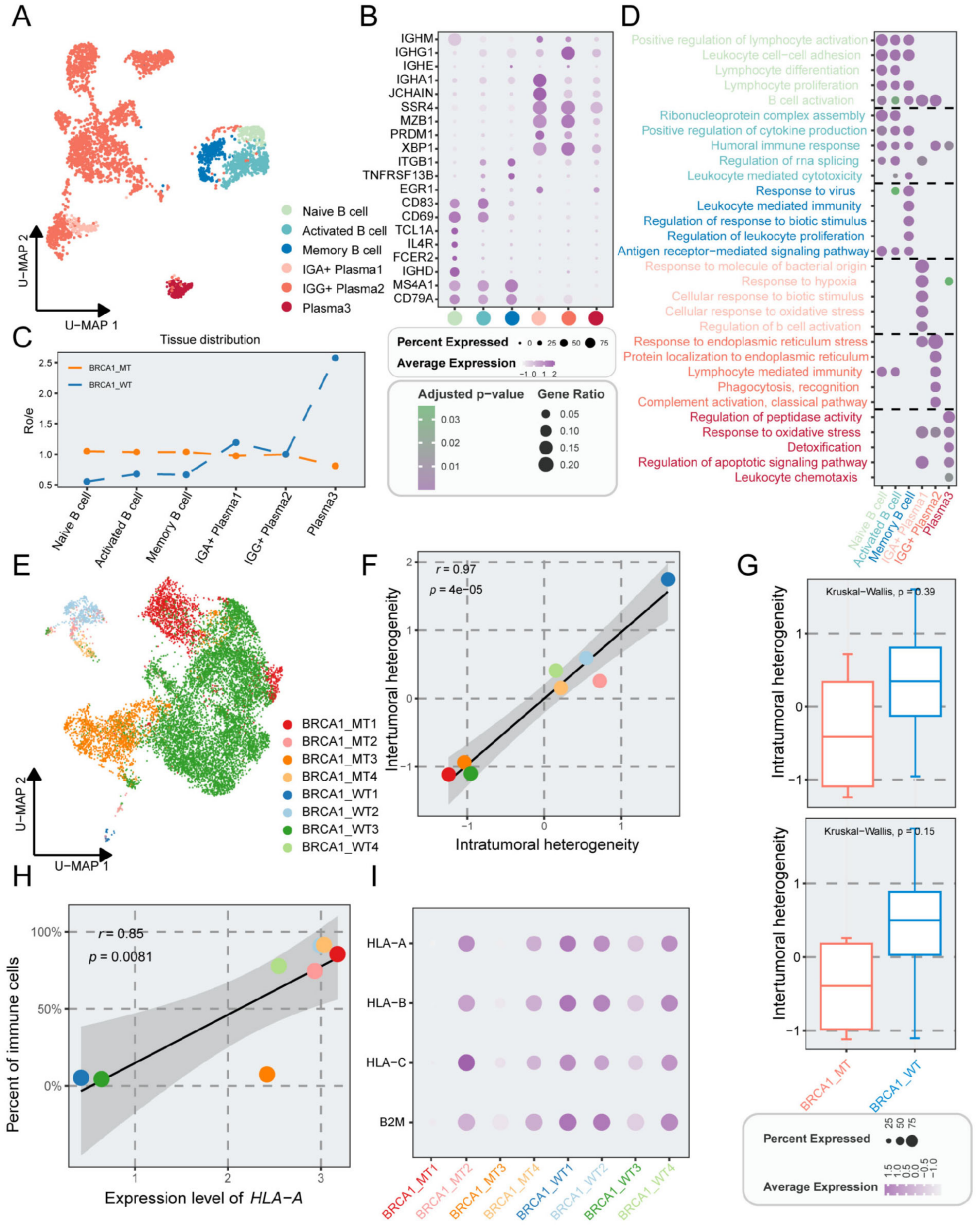
The properties of B cells and epithelial cells in TNBC patients with or without BRCA1 mutation. **(A)** UMAP plot showing the distribution of B cells, colored by cell subpopulations. **(B)** Average gene expression of selected marker genes for B cells. **(C)** The tissue prevalence for each cell subcluster via Ro/e analysis. **(D)** Top five biological process terms of each B subcluster via GO analysis. **(E)** UMAP plot of epithelial cells, colored by sample. **(F)** Scatter plot showing the correlation of intratumoral heterogeneity and intertumoral heterogeneity, colored by sample. **(G)** Comparison of intratumoral and intertumoral heterogeneity between the BRCA1-WT and BRCA1-MT groups. Student’s t-test. **(H)** Scatter plot showing the correlation of the expression level of HLA-A and percent of immune cells, colored by sample. **(I)** Average gene expression of HLA-associated genes among epithelial subpopulations.

Next, we investigated the B cell subclusters in TNBC. The B cells were subdivided into six subpopulations based on canonical marker genes (**Figures 4A, B**). Utilizing Ro/e analysis, we found that plasma3 cells were preferentially distributed in the BRCA1-WT group (**Figure 4C**). FDCSP, one marker gene of this subcluster, was associated with the activation and differentiation of B cells (**Supplementary Figure S6A**). For delving into the complexity of function within B cells, GO analysis was employed. Plasma3 cells were enriched in ROS regulation pathways, such as response to oxidative stress, and detoxification, which served as the signal regulator of B cells (36) (**Figure 4D**).

### Heterogeneity of gene expression and a special pro-metastasis subcluster in epithelial cells

Having evaluated immune cell heterogeneity, our attention subsequently shifted to epithelial cells. Tumor epithelial cells were distinguished from non-malignant epithelial cells via the CopyKAT package and were further divided into 19 cell subpopulations (**Supplementary Figures S6B, C**). Compared to the immune and stromal cells, significant differences were observed in epithelial cells across distinct specimens, revealing substantial intrasample heterogeneity (**Figures 4E**, **1B**). The expression of signature for distinct tumor subsets was identified (**Supplementary Figure S6D**). Intriguingly, Epi15, characterized by high expression of ISG15 was found mainly in the BRCA1-WT4 specimen. ISG15 is regarded as a critical proto-oncoprotein that enhances the proliferation and metastasis of TNBC via inhibiting ubiquitin pathway (37, 38), and may also mediate tumor immunity via the JAK/STAT signaling pathway (39, 40).

We assessed the tumor heterogeneity scores across TNBC samples, demonstrating a significant positive correlation between intra- and inter-tumoral heterogeneity (r = 0.97, p < 0.001) (**Figure 4F**). Furthermore, marginally elevated heterogeneity scores were found in the BRCA1-WT group when compared to the BRCA1-MT group (**Figure 4G**). Intriguingly, the expression levels linked to human leukocyte antigen class I (HLA-I) displayed inconsistency across TNBC samples, implying potential deficits in the antigen-presenting capabilities of tumor cells (**Figure 4I**). Specifically, the mean expression level of HLA-A correlated positively with the percent of immune cells (**Figure 4H**). Despite higher immune cell proportions in BRCA1-MT samples, there were significant variations among these samples (**Figure 4I**).

### BRCA1-driven CAF reprogramming and clinical outcomes in TNBC

**Figure 5:**
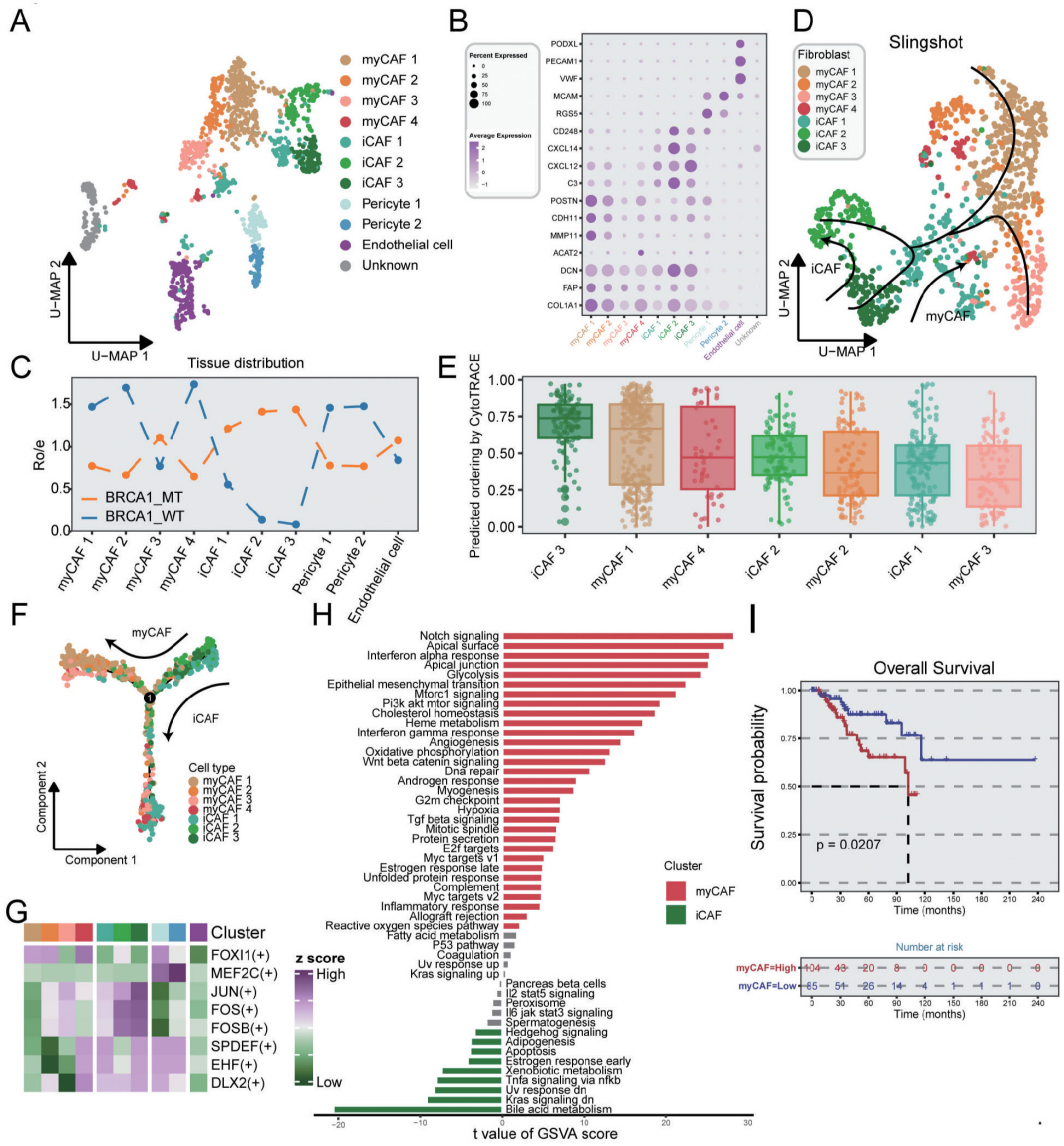
Characterization of stromal cells in TNBC samples. **(A)** UMAP plot showing the distribution of stromal cells, colored by cell subpopulations. **(B)** Average gene expression of selected marker genes for stromal subclusters. **(C)** The tissue prevalence for each subcluster estimated via Ro/e analysis. **(D)** The trajectory analysis of CAF subclusters via slingshot, colored by cell subpopulations. **(E)** Boxplot indicating the differential potential of CAF subclusters using CytoTRACE algorithm, colored by cell subpopulations. **(F)** The developmental trajectory of CAF subclusters utilizing the monocle2 algorithm, colored by cell subpopulations. **(G)** Heatmap showing the differential activated TFs in each stromal subcluster. **(H)** Bar plot indicating the differentially activated pathways compared between myCAF and iCAF clusters. **(I)** K-M survival analysis of patients from the METABRIC dataset with low and high infiltration abundance of the myCAF subgroup. Log-rank test.

Stromal cells in TME exhibited obvious functional and phenotypical heterogeneity and comprised multiple subsets. In stromal cells, we identified five cell types with distinct functional features, including myofibroblastic CAFs (myCAFs), inflammatory-like CAFs (iCAFs), pericytes, endothelial cells, and unknown cells (**Figure 5A**). The unknown subset was regarded as double cells and excluded from the subsequence analysis. As shown in **Figure 5B** and **Supplementary Figure S7**, myCAFs were characterized by the high expression of POSTN and ACAT2, which showed immune-suppressive and pro-invasive TME (41), while iCAFs exhibited elevated cytokine-related gene expression, such as CXCL12, and CXCL14. It was revealed by distribution analysis that myCAFs were primarily gathered in the BRCA1-WT group, while iCAFs were mainly enriched in the BRCA1-MT group (**Figure 5C**). Notably, the iCAF3 subset, highly expressing MEG3, showed a similar distribution in the two groups. As previously characterized, MEG3+ CAFs could modulate metalloprotease-associated gene expression, including MMP1, MMP3, and MMP9, which was the signature of pre-CAFs (42, 43). Thus, iCAF3 subset was regarded as the original of CAFs, as evidenced by CytoTRACE (**Figure 5E**). Monocle analysis further demonstrated that the iCAF3 subset differentiated into myCAFs and iCAFs along separate pathways, as corroborated by Slingshot (**Figures 5D, F**). These findings suggested that BRCA1 mutation status might be a potential key driver of CAF differentiation direction. Using SCENIC analysis, iCAFs exhibited the activation of some essential TFs, such as JUN, FOSB, and FOS (**Figure 5G**), reflecting high response probability to ICIs (44). Furthermore, GSVA analysis revealed that the TNFα pathway was preferentially activated in iCAFs (**Figure 5H**). Taken together, we deduced that BRCA1 mutation mediated homologous recombination deficiency (HRD), thus promoting the activation of the TNFα pathway via modulating the JUN and FOS expression, and ultimately induces the iCAF phenotype.

For myCAFs that were enriched in the BRCA1-WT group, the TGF-β signaling pathway was found to promote the myCAF transformation. GSVA analysis indicated that epithelial-mesenchymal transition and apical junction pathways were also observed in myCAFs, suggesting that myCAFs might facilitate cancer metastasis (**Figure 5H**). We employed ssGSEA to assess the clinical significance of signature genes from myCAF in TNBC patients, demonstrating a correlation between elevated levels of this subset and poor prognosis (**Figure 5I**). In summary, our findings suggested that BRCA1 mutations could induce CAF reprogramming within the TME, thereby mediating distinct clinical outcomes in TNBC.

### Cell–cell interactions of CXCL signaling sent primarily by the iCAFs and CD99 signaling sent primarily by the myCAFs

**Figure 6:**
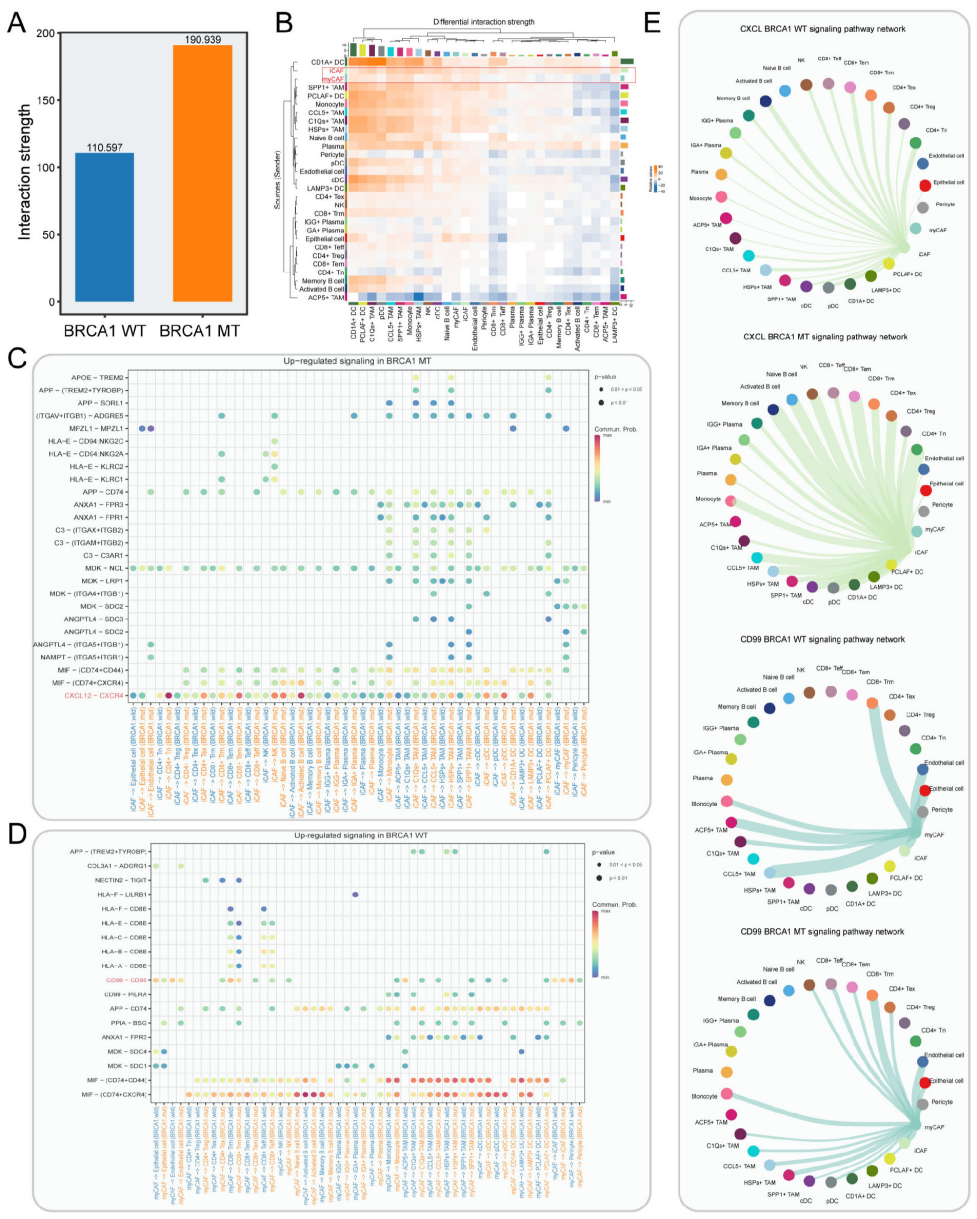
Cell-cell interaction network in BRCA1-WT and BRCA1-MT samples. **(A)** Bar plot showing the interaction strength between BRCA1-WT and BRCA1-MT samples. **(B)** Heatmap indicating the differential interaction strength of cell-cell interaction network between BRCA1-MT and BRCA1-WT samples. The bar represents the accumulated strength of interactions that were sent (right) or received (top) by each cell type. **(C, D)** The elevated receptor/ligand interactions of CAFs with other cell subsets in the BRCA1-MT **(C)** and BRCA1-WT samples **(D)**. **(E)** The inferred CXCL and CD99 signaling pathway network. Edge width denotes the communication probability.

To dissect the intercellular interaction network in TNBC, CellChat was performed. Unsurprisingly, the BRCA1-MT group had more interaction strengths compared to the BRCA1-WT group, reflecting that BRCA1 mutation drove complex communication networks (**Figure 6A**). As shown in **Figure 6B**, the iCAFs exhibited the elevated strength of outgoing signals in the BRCA1-MT, while myCAFs had the increased strength of outgoing signals in the BRCA1-WT group. Furthermore, we identified the differential molecular crosstalk between CAF and other cell subpopulations (**Figures 6C, D**). Notably, the iCAF-mediated CXCL12-CXCR4 receptor/ligand pair was significantly enhanced in the BRCA1-MT group, while myCAF-mediated CD99-CD99 was strikingly increased in the BRCA1-WT group. More specifically, the receivers of increasing CXCL12 signaling network were observed to involve nearly all immune cell types in the BRCA1-MT group, reflecting its central role in driving immune regulation (45). For the CD99 signaling network, we observed decreased crosstalk communications between myCAFs and other immune cell types in the BRCA1-WT group, indicating its role in inhibiting immune infiltration (**Figure 6E**).

### Multi-scale integrated analysis pinpointed ISG15 as a critical myCAF-associated gene

**Figure 7:**
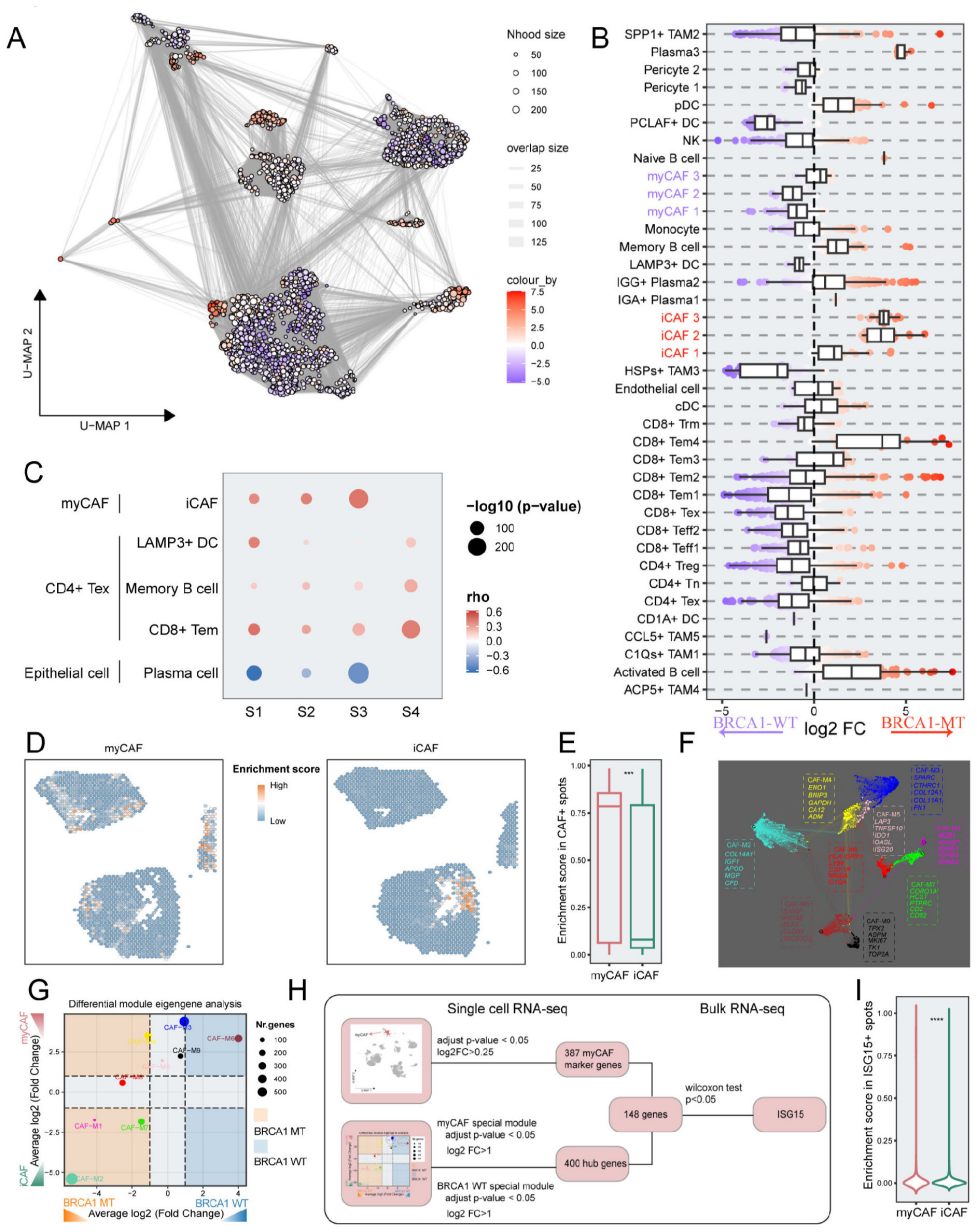
The identification of the key genes via integrated multi-scale analysis. **(A)** Neighborhood graph of immune and stromal cells utilizing Milo differential abundance testing. Nodes represent neighborhoods from the cell populations. Colors indicate the log2-fold change between BRCA1-WT and BRCA1-MT patients. Neighborhoods that enriched in BRCA1-MT patients are shown in red. Neighborhoods deleted in BRCA1-MT patients are shown in blue. **(B)** Beeswarm and boxplots of cell subpopulations from immune and stromal cells based on Milo differential abundance testing. **(C)** The correlation of spatial position among a subset of cell clusters in each sample. Spearman correlation. **(D)** Spatial distribution of enrichment scores for myCAF and iCAF. **(E)** Boxplot showing the difference of enrichment scores between myCAF and iCAF in CAF+ spots. **(F)** Gene co-expression network of TNBC. Each dot represents a single gene, colored by the gene module. The dot size is scaled by the eigengene-based connectivity (kME) of the gene. The top five genes of each module are shown. **(G)** Phenotype-specific gene modules were identified via differential module eigengene analysis. The significance threshold was set to log2FC (fold change) > 1 and adjusted p-value<0.05. **(H)** A flowchart depicting the selection pipeline of key genes based on multi-scale data, including scRNA-seq and bulk RNA-seq. **(I)** Comparison of enrichment scores between myCAF and iCAF in ISG15+ spots. Wilcoxon rank-sum test. *** p<0.001, **** p<0.0001.

We reasoned that finer-resolution changes in cell subpopulations may be masked when analyzing the relative frequencies of broad cluster-based cell type identifications. To obtain subtler differences of cell subsets within TME, we grouped the cells into “neighborhoods” through k-nearest neighbor clustering, as implemented via the milo analysis framework. Differential abundance analysis of cell neighborhoods revealed that BRCA1-WT patients exhibited significant enrichment for myCAFs and BRCA1-MT patients were characterized by the enrichment of iCAFs (**Figures 7A, B**). To further elucidate the interactions among cell types at the spatial scale, four BRCA1-WT TNBC samples were included in this study. For every spot within the spatial data, we calculated the enrichment scores of each cell type, and correlation analyses were performed in each spatial RNA-seq sample (**Figure 7C**). The enrichment scores of myCAFs and iCAFs exhibited a significant positive correlation. In contrast, myCAFs and iCAFs demonstrated a clear spatial exclusivity (**Figure 7D**). These results reflected the homogeneity and heterogeneity within CAF subgroups. In addition, we observed a high degree of colocalization among CD4+ Tex, Bm, LAMP3+ DC, and CD8+ Tem, suggesting the presence of tertiary lymphoid structures (**Supplementary Figure S8A**). The mutual exclusivity relationship was also observed between epithelial cells and plasma cells (**Supplementary Figure S8B**).

We examined the enrichment scores of CAF subclusters in specific CAF spots. It was evident that myCAFs had substantially increased enrichment scores, consistent with their primary role in the CAF phenotype atlas of BRCA1-WT samples (p < 0.001) (**Figures 7B**, **E**). To pinpoint functional gene modules associated with the myCAF subcluster in the BRCA1-WT group, we conducted a co-expression network analysis using hdWGCNA on CAF cells, obtaining nine gene modules (**Figure 7F**). Notably, both CAF-M3 and CAF-M6 were significantly linked to the BRCA1-WT phenotype and the myCAF subcluster (log2FC > 1, p < 0.05) (**Figure 7G**). Employing a multi-scale identification framework, ISG15 was regarded as a crucial gene within myCAFs from BRCA1-WT patients, as devised in **Figure 7H**. Next, the association between ISG15 expression and myCAFs was also validated through spatial RNA-seq data. In spots expressing ISG15, the enrichment scores of myCAFs substantially exceeded others (p < 0.0001) (**Figure 7I**).

### Construction of an ISG15-driven predictive system for ICI response

**Figure 8:**
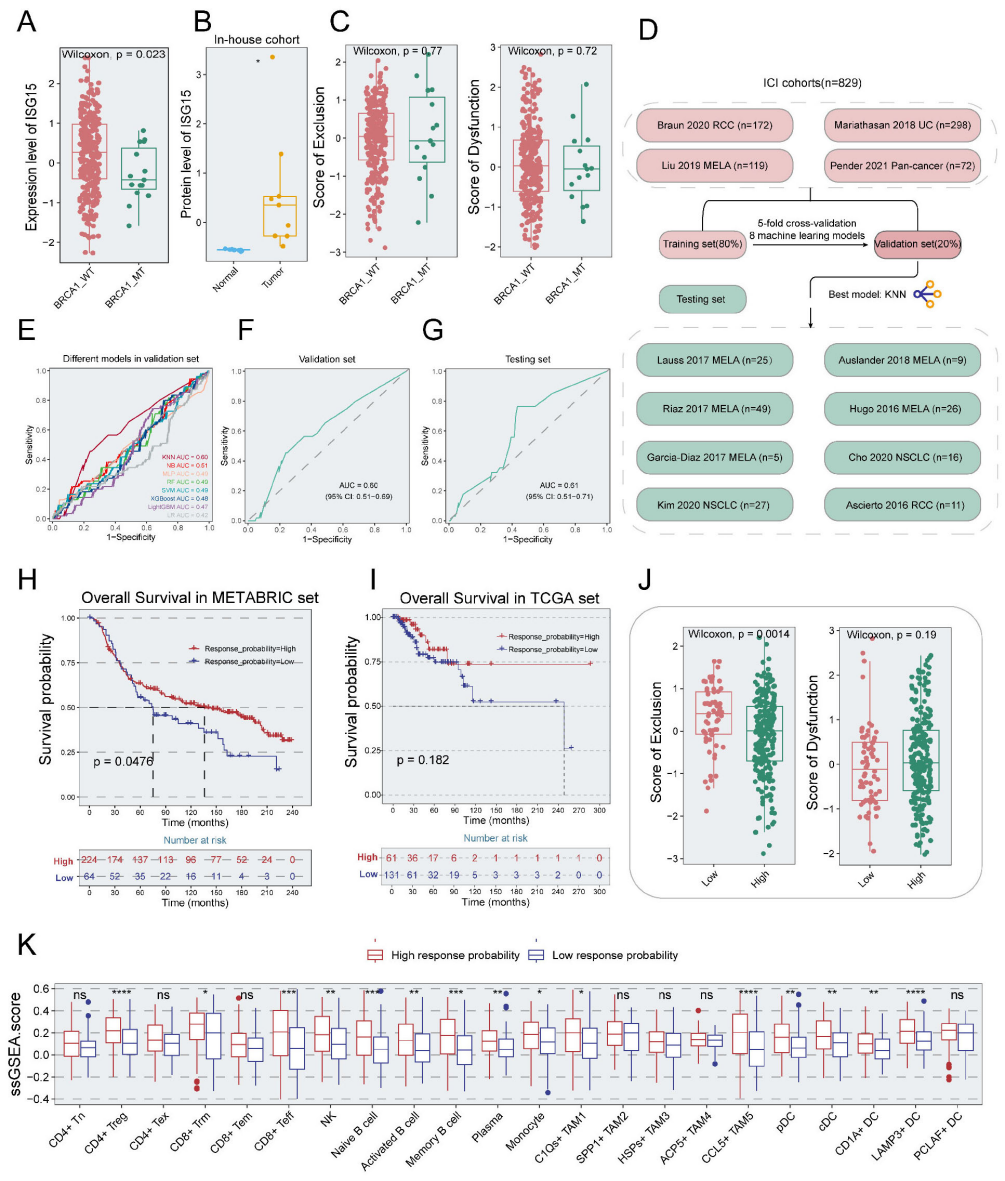
Establishment of a predictive model for immunotherapy response. **(A)** Boxplot showing the difference of ISG15 expression levels between BRCA1-WT and BRCA1-MT groups in TNBC specimens from METABRIC dataset. Wilcoxon rank-sum test. **(B)** Higher expression of ISG15 occurred in tumor tissue compared to normal tissue from the in-house proteomics cohort. T-test. **(C)** Boxplot indicating the difference of immune dysfunction (left panel) and exclusion scores (right panel) between BRCA1-WT and BRCA1-MT groups in TNBC specimens from METABRIC dataset. Wilcoxon rank-sum test. **(D)** workflow of developing the ISG15-associated model via machine learning algorithms. **(E)** ROC curves indicating the difference of multiple models in the validation set. **(F)** ROC curve of KNN model in the validation set. **(G)** ROC curve of KNN model in the testing set. **(H, I)** K-M survival analysis based on the response probability from KNN model in the METABRIC dataset **(H)** and TCGA dataset **(I)**. Patients in the low response probability group had poor survival. Log-rank test. **(J)** Boxplot indicating the difference of immune exclusion (left panel) and dysfunction scores (right panel) between high and low response probability groups in TNBC specimens from METABRIC dataset. Wilcoxon rank-sum test. **(K)** The difference in ssGSEA scores of immune cell types between the high and low response probability groups in TNBC specimens from METABRIC dataset. Wilcoxon rank-sum test. * p<0.05, ** p<0.01, *** p<0.001, **** p<0.0001.

The expression level of ISG15 was significantly upregulated in the BRCA1-WT group compared to the BRCA1-MT group (p< 0.05) (**Figure 8A**). Given this, we reasoned that a subset of patients within the BRCA1-WT group, who exhibit BRCA1-like characteristics, may influence ISG15 expression. Thus, the TNBC samples were split into BRCA1-like and non-BRCA1-like groups as depicted by Chen et al (46). The comparison of ISG15 expression showed that the elevation of ISG15 expression was observed in the BRCA1-like group compared to the non-BRCA1-like group (**Supplementary Figure S9A**). We next evaluated the genetic vulnerabilities in eight TNBC cell lines via the CRISPR gene essentiality data extracted from the DepMap database. The result suggested that ISG15 was the most essential gene for SUM229PE cells, one of the BRCA1-WT cell lines (**Supplementary Figure S9B**). Our proteomics cohort (in-house cohort) indicated a substantial upregulation of ISG15 expression in breast cancer tissues compared to normal tissues, a finding supported by paired comparisons in the GSE109169 cohort. (**Figure 8B**, **Supplementary Figure S9C**). In summary, our study indicated that ISG15 was significantly upregulated in breast cancer, however, its functional role in TNBC specimens with BRCA1 mutation remained elusive.

Next, we evaluated the relationship between immunotherapeutic response and BRCA1-related classification systems including BRCA1 mutation and BRCA1-like via the TIDE (Tumor Immune Dysfunction and Exclusion) algorithm. No significant association between the scores for dysfunction and exclusion and the BRCA1 status (p > 0.05) (**Figure 8C**). Exclusion score was significantly elevated in the BRCA1-like group relative to the non-BRCA1-like group, and a higher dysfunction score was observed in the non-BRCA1-like group (**Supplementary Figure S9D**). These results indicated that the classification system based on BRCA1 status showed poor performance for immunotherapeutic response, and the development of a novel predictive model is particularly necessary.

As previous research indicated, ISG15 played a key role in regulating the immune infiltration of the TME, and its upregulation could synergically augment the therapeutic efficacy of ICIs (47). Utilizing protein-protein interaction analysis, we identified 50 ISG15-associated genes (**Supplementary Figure S9E**). Logistic regression was conducted 1,000 times to select the robust predictive features for immunotherapeutic response, yielding 11 genes as the input features for developing the model. Based on the 12 cohorts with immunotherapeutic response information, we used eight machine learning to devise the predictive model. A 5-fold cross-validation approach was employed to streamline the model and the ROC value was utilized to evaluate the performance of models (**Figure 8D**). Comparison of the performance of eight predictive models in the validation set showed that the greatest AUC of 0.60 (95% CI: 0.51–0.69) occurred in the KNN model, which was utilized for subsequent analysis (**Figures 8E, F**). For the testing set, the KNN model had an AUC of 0.61 (95% CI: 0.51–0.71), as shown in **Figure 8G**. To further explore the clinical significance of the KNN model, survival analysis was performed on TNBC patients from the METABRIC cohort. Increased response probability was associated with favorable overall survival (p < 0.05) (**Figure 8H**). In the TNBC patients from the TCGA cohort, we observed a similar trend of overall survival (p = 0.182) (**Figure 8I**).

Unlike the classification systems of BRCA1 mutation and BRCA1-like, the high response probability group exhibited significantly lower exclusion scores (p < 0.05) and slightly higher dysfunction scores (p = 0.19), as depicted in **Figure 8J**. Utilizing ssGSEA, we found that elevated immune infiltration scores were observed in the high response probability group relative to the low response probability group (**Figure 8K**).

The global feature importance for the KNN model was assessed using the DALEX package, identifying PCNA, STAT1, IFI6, and OAS1 as the four most significant genes (**Supplementary Figure S9F**). PCNA, a key immunological checkpoint molecule, is suggestive of a favorable response to NK cell-based immunotherapy (48). The stability of PCNA was augmented by ISG15 modification (ISGylation), substantiating the model’s validity in predicting immune therapy responses (49). ISG15 and STAT1 collectively activated IFN-related pathways, which promoted the release of IFNγ within immune cells, ultimately modulating accumulated immune infiltration (50). Furthermore, the expression of IFI6 was markedly positively associated with the abundance of B and T cells within TME, indicating a key role in immune regulation (51). The RNA sensor OAS1 and its upregulation responded to the amplification of IFN-1 and immune suppression genes, as evidenced by the partial elevation of immune suppressive cells in the high response probability group (50). Taken together, our predictive model has the potential to accurately identify TNBC patients who benefit from ICI therapy compared to the prediction based on BRCA1 mutation status.

## Discussion

TNBC is considered a unique subtype of breast cancer with an unfavorable prognosis, known by high heterogeneity and lacking effective therapeutic targets (52). Notably, around half of TNBC patients exhibit BRCA1 mutation (13). Recent findings have indicated that BRCA1-mutant TNBCs are potentially responsive to ICI treatments owing to their high immunogenicity and mutational burden (15). Nevertheless, understanding of the TME in BRCA1-mutant TNBC remains unclear. In this study, we leveraged scRNAseq, spatial transcriptome, and bulk RNAseq data to comprehensively unravel the TME landscape in the BRCA1-MT TNBC, compared to the BRCA1-WT TNBC. **Supplementary Table S4** provides a clearer summary of the phenotypic differences across epithelial, immune, and stromal compartments by integrating the key findings from multi-omic analyses. Our analysis revealed that immune and stromal components were enriched in the BRCA1-MT group, which was regarded as “hot” tumor. In contrast, the BRCA1-WT group was primarily composed of tumor cells, in line with the features of “cold” tumor (53). Through subpopulation analysis, we delved deeper into the cellular heterogeneity of the TME in these two types of TNBC, providing new insights into TME distributions driven by BRCA1 mutation. Our research also found that BRCA1 mutation drove the distinct differential direction of CAFs into iCAF or myCAF. Through Cell communication analysis, we identified iCAF-mediated CXCL12-CXCR4 and myCAF-mediated CD99-CD99 signaling networks to offer new therapeutic strategies for both two types of TNBC. Subsequently, based on multi-scale data analysis, we identified ISG15 as a key immune molecule and developed an ISG15-associated predictive system to predict responses to ICIs.

Our study performed a comprehensive comparison of the TME landscapes in BRCA1-MT and BRCA1-WT TNBC. The BRCA1-WT group exhibited the enrichment of elevated exhausted but enhanced cytotoxic T cells, alongside tolerant DC. Spatial transcriptomics revealed positive correlations between Tex, Treg, and LAMP3+ DCs. Notably, the increased CXCL13 expression in the CD4+ Tex subset induced the formation of tertiary lymphoid structure (54), suggesting enhanced cytotoxic activity in the BRCA1-WT group. This group also displayed robust expression of HLA-related molecules, indicating the immunotherapeutic response probability (55). Unfortunately, the dominant tumor cells and myCAFs increased the stiffness of TME, suggesting a poorer immune response. In contrast, BRCA1 mutation was associated with higher levels of immune infiltration, suggesting effective immune responses (56). BRCA1 mutation accumulated more tumor mutational burdens and induced the elevation of neoantigen, thereby broadly activating T cells in the TME. Mechanistically, BRCA1 mutation-mediated DNA damage enhanced the activation of NF-κB pathway signaling, thus promoting inflammation and immune cell infiltration (57, 58). Despite accumulated neoantigen production in the BRCA1-MT group, the heterogeneous expression of HLA-related genes within tumor cells may impair immune cell recruitment and ICI response in this group.

As critical components of the TME, TAMs participated in various processes including tumor growth, angiogenesis, immune regulation, metastasis, as well as chemotherapy resistance. Through reclustering of the myeloid lineage, we identified five unique TAM subpopulations with distinct functional phenotypes. Mirroring previous reports, SPP1+ TAMs exhibited the high expression of SPP1, CCL20, and angiogenesis-associated genes, notably VCAN and VEGFA, while C1Qs+ TAMs expressed C1Qs, APOE, and SLC40A1. Complement C1q, including C1QA/B/C, improves phagocytosis but inhibits inflammation for macrophage (59). Moreover, three distinct TAM subclusters were identified. HSPs+ TAM was characterized by the elevated expression of HSPA1A, HSPA1B, and HSPB1, corroborating prior studies that HSPs cause the production of tolerogenic TAMs (60). Mechanically, augmented HSPs also potentiate breast cancer metastasis via the EMT process (61). ACP5+ TAMs were linked to overexpression of metalloenzyme-related genes encompassing ACP5, MMP9, and CTSP, increasing the metastatic potential of TNBC cells (62). CCL5 derived from TAMs promotes tumor invasion, metastasis, and the self-renewal of BCSCs (63). These TAM subclusters mentioned above contributed to the aggressive and immune-tolerant characterization of BRCA1-WT TNBC. Nevertheless, BRCA1-MT TNBC showed enhanced angiogenesis and boosted innate phagocytic activity. Additionally, our study also demonstrated that macrophage characteristics based on vascular and phagocytic phenotypes were accurately captured rather than the traditional M1/M2 classification.

In the TME, CAF is the most prominent stromal cell type with extensive cellular interactions. Utilizing Ro/e and global differential abundance analyses, myCAFs were predominantly found in the BRCA1-WT group, whereas iCAFs were prevalently enriched in the BRCA1-MT group. Further, multiple pseudotime analyses identified a novel origin subpopulation of CAFs (iCAF3), termed pre-CAF. The CAF subpopulations showed a complex relationship of homology but spatial exclusion, underscoring the CAF heterogeneity within TME. Through differential activation of TFs and enrichment pathways, we dug into the underlying molecular mechanism of BRCA1 influencing the differentiation of pre-CAFs. BRCA1-mediated DNA damage stimulated the TNFα signaling pathway via activating TFs including FOS and JUN, thereby mediating iCAF transformation. In contrast, an enhanced TGF-β signaling pathway in the BRCA1-WT group induced myCAF differentiation. Unintriguingly, CellChat analysis underscored more frequent cellular interactions occurred in the BRCA1-MT group, marked by an abundance of non-tumor cells. The extensive activation of the CXCR4-CXCL12 signaling network is observed within the “hot” TME, while the CD99-CD99 signaling network is prevalent across the “cold” TME (53). Our cell communication analysis not only supported these observations but also emphasized the crucial role of CAFs in TNBC.

ISG15 encodes the ISG15 protein, which is involved in multiple cell processes, encompassing cell motility and tumor invasion (64). Prior studies suggest that ISG15 may serve as a promising immunomodulatory to modulate the TME towards inhibiting tumor direction (65). Employing a multi-scale framework, ISG15 was pinpointed as a crucial gene in myCAFs of BRCA1-WT patients. With the aid of spatial transcriptomics and proteomics data, we confirmed that ISG15 is highly expressed and predominantly co-located with the myCAF subcluster in breast cancer, underlining its significant role in pro-tumor. Interestingly, despite previous reports of its elevated expression in BRCA1 mutant TNBC cell lines (66), we found that ISG15 was a signature gene of the myCAF subset in the BRCA-WT group. Employing the BRCA1-like classification system, we noted high ISG15 expression in the BRCA1-like category. Additionally, TNBC cell line data from the DepMap database indicates that ISG15 ranks as the top essential gene for survival in the TNBC-WT cell line (SUM229PE). In line with this paradoxical phenotype, ISG15 is elevated both in stromal and cancer cells (67).

Machine learning has been widely utilized in medical research for decoding the TME and predicting therapeutic responses. In light of the essential role of ISG15 in tumor immunoregulation, we constructed an ISG15-associated predictive system for ICI response leveraging our proposed machine learning framework. This model had good performance for evaluating immune cell content in the TME and forecasting immunotherapy effectiveness. As ISG15 acts as a double-edged sword in immunoregulation, pro-tumorigenic immune cells were inevitably increased, leading to the decreased performance of this model. Despite BRCA1 status are not significantly correlated with immune cell content, BRCA1 mutation-mediated TME still exhibits more anti-tumor activity. Thus, the combination of BRCA1 mutation status with our predictive model could enhance the accuracy in identifying patients who benefit from ICI therapy.

Although our study provides significant insights into the TME heterogeneity between the BRCA1-WT group and the BRCA1-MT group from TNBC patients, several limitations should be further explored. To comprehensively delineate the TME differences between the two groups from the spatial transcriptome perspective, spatial RNAseq data on BRCA1 mutation should be collected and analyzed. Furthermore, although the predictive system was developed from multiple ICI cohorts, it has not yet been validated in TNBC cohorts treated with ICIs. Moreover, our researches are supported solely by multi-scale transcriptome data analysis, the underlying molecular mechanisms require further elucidation.

## Conclusions

In conclusion, our multi-scale transcriptomic analysis of BRCA1 status in TNBC provides new insights into their role in structuring the TME. Furthermore, ISG15 was highlighted as a potential immunoregulatory marker associated with TME alterations between the two groups, warranting further investigation into its function and therapeutic relevance. Moreover, the study constructs a predictive system to identify precisely the TNBC patients for clinical benefits.

## Data Availability

The single-cell RNA sequencing data used in this study were obtained from the Gene Expression Omnibus (GEO, https://www.ncbi.nlm.nih.gov/geo/) under accession number GSE161529. Spatial transcriptomics data are available at Zenodo (https://zenodo.org/record/4739739). Bulk transcriptomic, somatic mutation, and clinical data for TNBC were retrieved from The Cancer Genome Atlas (TCGA, https://portal.gdc.cancer.gov/) and the METABRIC cohort via cBioPortal (https://www.cbioportal.org/study/summary?id=brca_metabric). The external validation dataset was obtained from GEO (GSE109169). Immune checkpoint inhibitor cohorts were integrated from GEO (GSE135222, GSE78220, GSE126044, GSE67501, GSE115821, GSE96619, GSE100797, GSE91061), the IMvigor210CoreBiologies R package, Genome Sciences Centre (https://www.bcgsc.ca/downloads/immunoPOG/), and two published studies (DOI: 10.1038/s41591-020-0839-y , 10.1038/s41591-019-0654-5 ). Proteomic data generated in this study were acquired via high-sensitivity label-free LC-MS/MS and processed using Proteome Discoverer 2.4 with a 1% FDR threshold; detailed methods are provided in the Supplementary Materials. The raw proteomic data are available from the corresponding author upon reasonable request.
