# Supplementary material for: Resolving tumor microenvironment heterogeneity to forecast immunotherapy response in triple-negative breast cancer through multi-scale analysis
Source: Front Oncol. 2025 Aug 19;15:1538574. doi: 10.3389/fonc.2025.1538574 (PMC12401915; doi:10.3389/fonc.2025.1538574)
Supplement: Supplementary file 1 [file DataSheet1.doc]

**Supplementary Methods**

**High-sensitivity label-free quantitative proteomics analysis**

Total protein extraction

Take out the samples in the frozen state and put them on ice. Add an appropriate amount of protein lysate (8 M urea, 1% SDS), which contains protease inhibitor to inhibit protease activity. The mixture was treated by ultrasound for 2 min at a low temperature, following splitting for 30 min. After centrifugating at 12000g at 4°C for 30min, the concentration of protein supernatant was determined by the Bicinchoninic acid (BCA) method by BCA Protein Assay Kit (Pierce, Thermo, USA). Protein quantification was performed according to the kit protocol.

Protein reductive alkylation and digestion

Take protein samples 100 μg and add TEAB (Triethylammonium bicarbonate buffer), of which the final concentration of TEAB is 100 mM. Then add TCEP (tris (2-carboxyethyl) phosphine) to the final concentration of 10 mM and react for 60 min at 37 °C. Following add IAM (Iodoacetamide) to the final concentration of 40 mM and react for 40 min at room temperature under dark conditions. Add a certain percentage (acetone: sample v/v = 6:1) of pre-cooled acetone to each sample and settle for 4 h at -20 °C. After centrifugating for 20 min at 10000 g, the sediment was collected, followed by adding 100 µL 100mM TEAB solution to dissolve. Finally, the mixture was digested with Trypsin overnight at 37 °C added at a 1:50 trypsin-to-protein mass ratio.

Peptide desalination and quantification

The peptides were vacuum dried, then resusoended with 0.1% TFA. Samples were desalted with HLB, and vacuum dried. Peptide concentrations were determined by peptide quantitation kit (Thermo, Cat.23275). Loading buffer was added to each tube to prepare samples for mass spectrometry analysis, and the concentration of each sample was 0.25 µg/µL.

LC-MS/MS analysis

Trypsin-digested peptides were analyzed by an EASY nLC-1200 system (Thermo, USA), coupled with a Q Exactive HF-X Hybrid Quadrupole-Orbitrap mass spectrometer (Thermo, USA) at Majorbio Bio-Pharm Technology Co. Ltd. (Shanghai, China). In brief, the C18-reversed-phase column (75 μm×25 cm , Thermo, USA) as equilibrated with solvent A (2% ACN with 0.1% formic acid) and solvent B ( 80% ACN with 0.1% formic acid). The peptides were eluted using the following gradient: 0-56 min, 5%-23% B; 56-62 min, 23%−29% B; 62-63 min, 29%−38% B; 63−63:30 min, 38%−48% B; 63:30-64 min, 48%-100% B; and 64-90min, 100%-100% B. The tryptic peptides were separated at a flow rate of 300 nL/min. The Q Exactive HF-X instrument was operated in the data-dependent acquisition mode (DDA) to automatically switch between full scan MS and MS/MS acquisition. The survey of full scan MS spectra (m/z 300-1500) was acquired in the Orbitrap with 60000 resolutions. The automatic gain control (AGC) target at 3e6 and the maximum fill time was 20 ms. Then the top 20 most intense precursor ions were selected into collision cells for fragmentation by higher-energy collision dissociation (HCD). The MS/MS resolution was set at 15000 (at m/z 100), the automatic gain control (AGC) target at 1e5, the maximum fill time at 50 ms, and dynamic exclusion was 18 seconds.

Protein identification

MS/MS spectra were searched using ProteomeDiscovererTM Software 2.4 software against UniProtKB/Swiss-Prot human database. The highest score for a given peptide mass (the best match to that predicted in the database) was used to identify parent proteins. The parameters for protein searching were set as follows: tryptic digestion with up to two missed cleavages, carbamidomethylation of cysteines as fixed modification, and oxidation of methionines and protein N-terminal acetylation as variable modifications. The false discovery rate (FDR) of peptide identification was set as FDR ≤ 0.01. A minimum of one unique peptide identification was used to support protein identification.
