# Supplementary material for: Resolving tumor microenvironment heterogeneity to forecast immunotherapy response in triple-negative breast cancer through multi-scale analysis
Source: Front Oncol. 2025 Aug 19;15:1538574. doi: 10.3389/fonc.2025.1538574 (PMC12401915; doi:10.3389/fonc.2025.1538574)
Supplement: Supplementary file 2 [file DataSheet2.docx]

**Supplementary Figures**


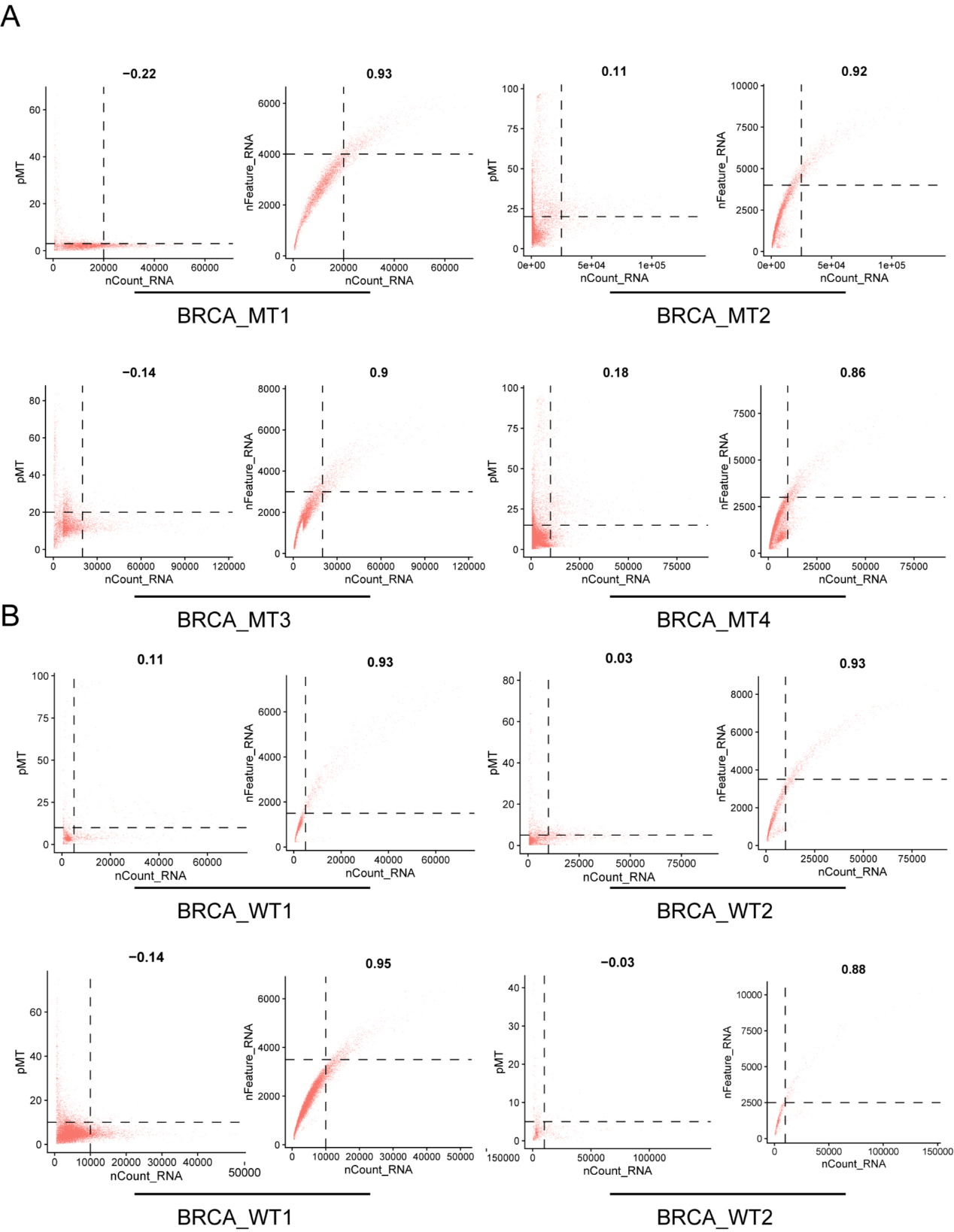


**Figure S1 Quality control for each sample in TNBC.** (A) Parameters of quality control for four BRCA1-MT patients (BRCA1-MT1-4) in this study, black dashed lines indicate the cut-off values for filtering. (B) Parameters of quality control for four BRCA1-WT patients (BRCA1-WT1-4) in this study, black dashed lines indicate the cut-off values for filtering.


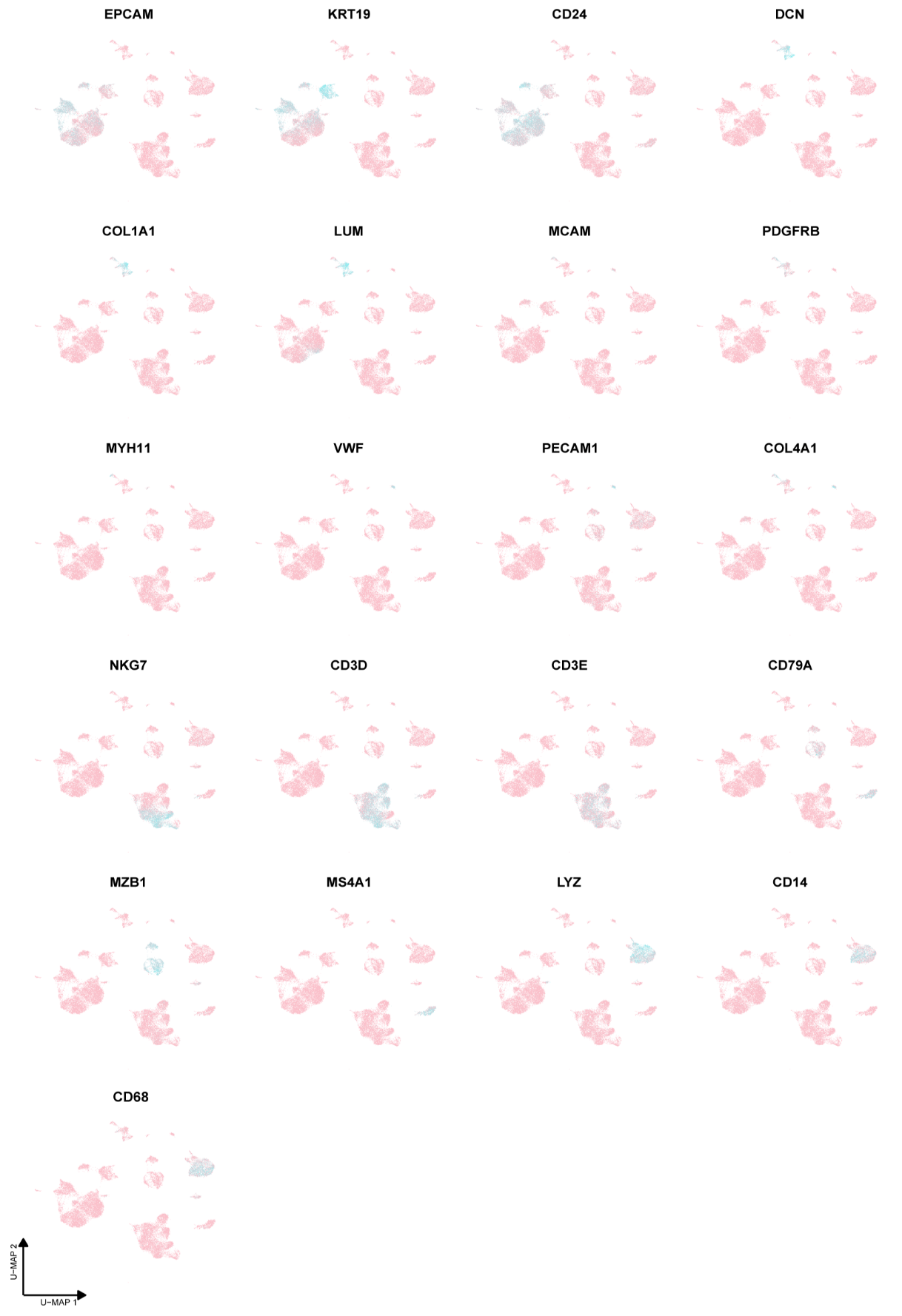


**Figure S2 Classical marker genes of each major cluster for 32,386 cells, visualized by feature plot.**


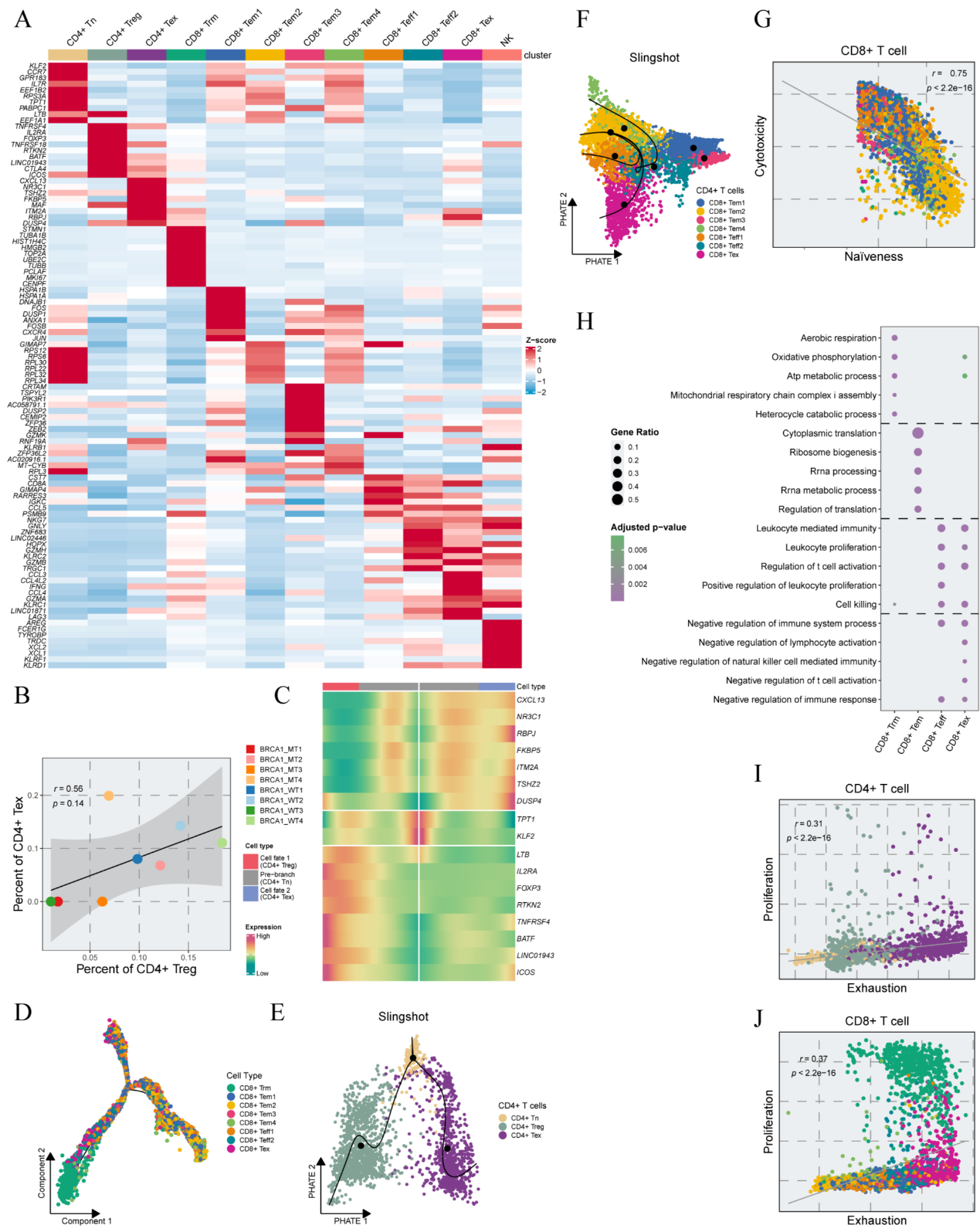


**Figure S3 Additional characteristics of T/NK clusters.** **(A)** Heatmap showing the top 10 differentially expressed genes for each T/NK subpopulation. **(B)** Correlation between proportions of Treg and Tex cells from CD4+ T cell cluster. Pearson correlation. **(C)** Differences in expression levels of marker genes along developmental trajectories of CD4+ T cells by BEAM analysis. **(D)** The developmental trajectory of CD8+ T cells, colored by cell subpopulations. **(E)** The trajectory analysis of CD4+ T cells via slingshot, colored by cell subpopulations. **(F)** The trajectory analysis of CD8+ Tem, Tex, and Teff cells via Slingshot, colored by cell subclusters from the associated cell subpopulations. **(G)** Correlation between naïveness and cytotoxicity score of CD8+ T cells, colored by cell subclusters. Pearson correlation. **(H)** Top five enriched biological process terms for each CD8+ T cell subpopulation via GO analysis. **(I-J)** Correlation between exhaustion and proliferation score of CD4+ (I) and CD8+ (J) T cells, colored by cell subpopulations. Pearson correlation.


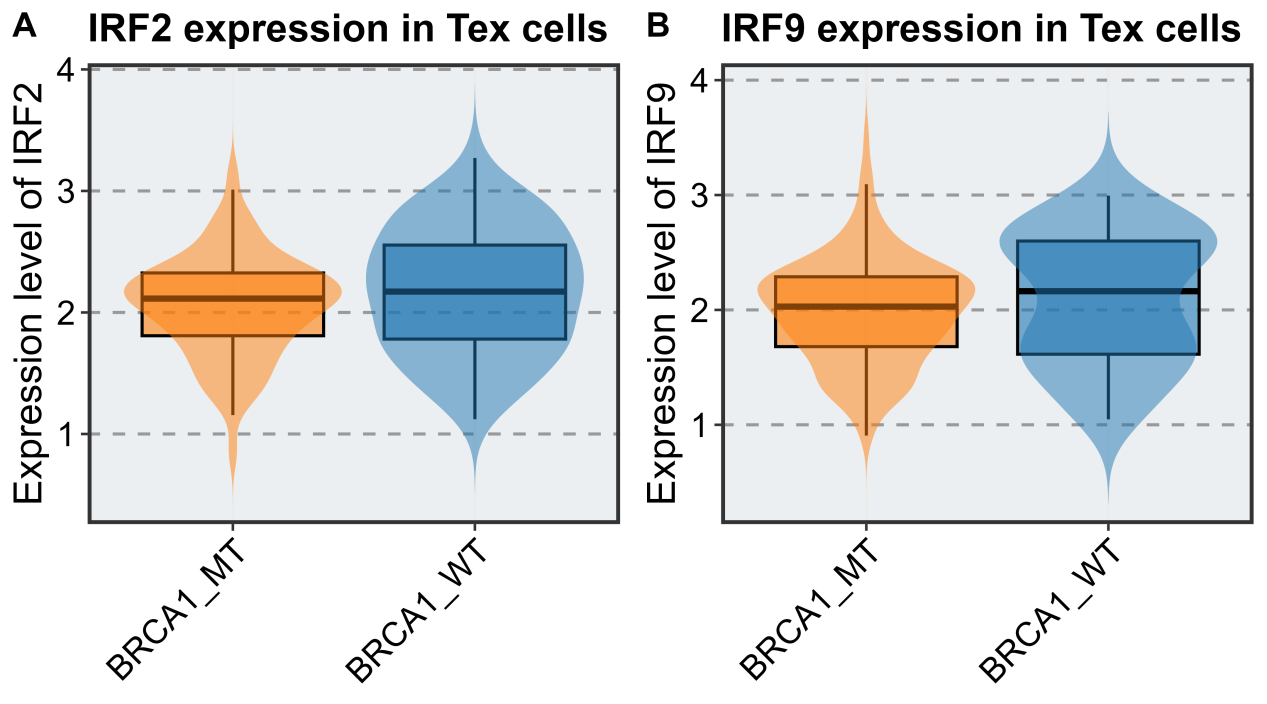


**Figure S4** Comparison of IRF2 **(A)** and IRF9 **(B)** gene expression levels in exhausted T cells between BRCA1-WT and BRCA1-MT groups.


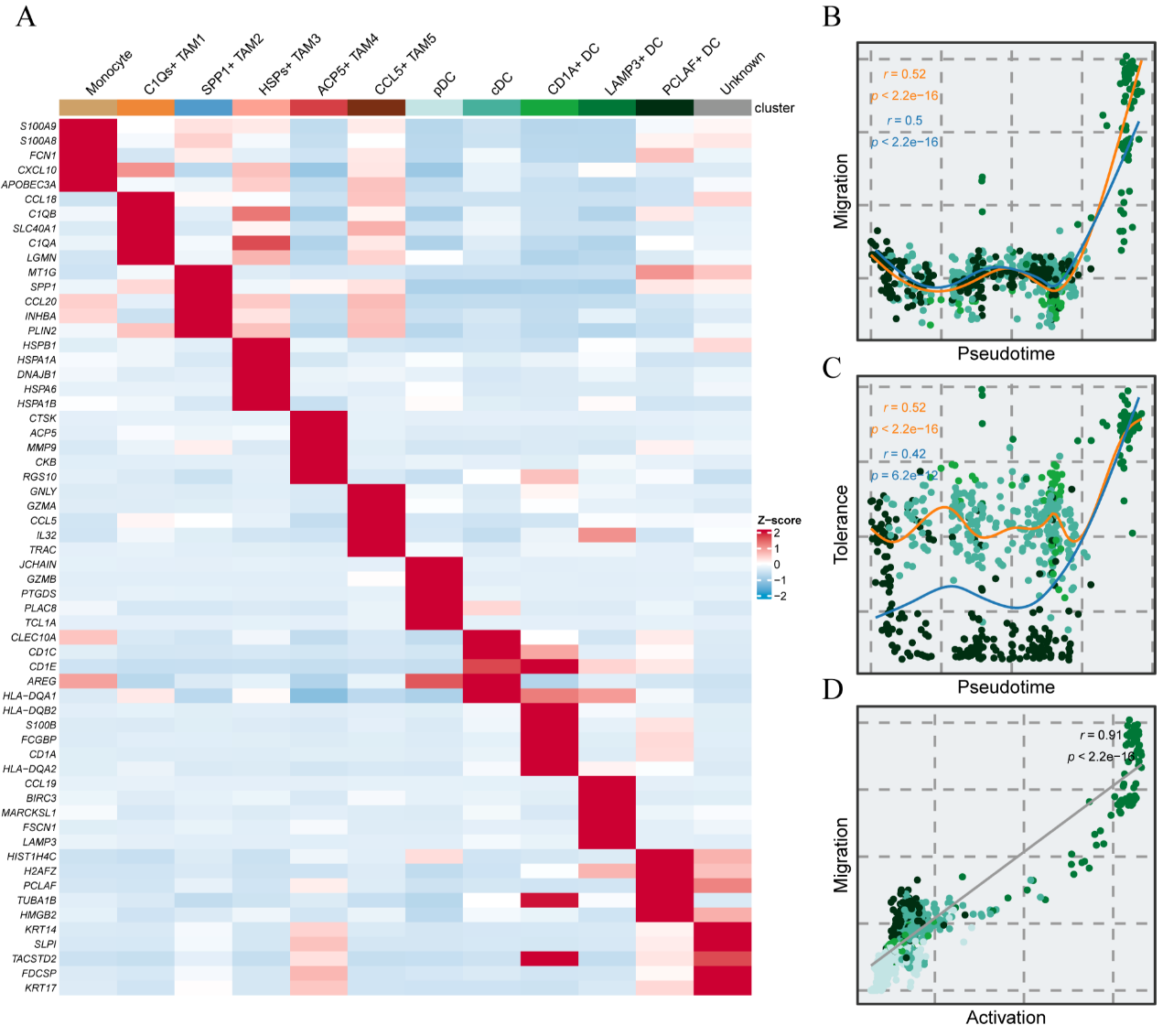


**Figure S5 Additional characteristics of myeloid clusters. (A)** Heatmap showing the top 10 differentially expressed genes for each myeloid subpopulation. **(B)** Scatter plot showing the correlation of pseudotime and migration score in DCs, colored by cell subpopulations. **(C)** Scatter plot showing the correlation of pseudotime and tolerance score in DCs, colored by cell subpopulations. **(D)** Scatter plot showing the correlation of migration and migration scores in DCs, colored by cell subpopulations.


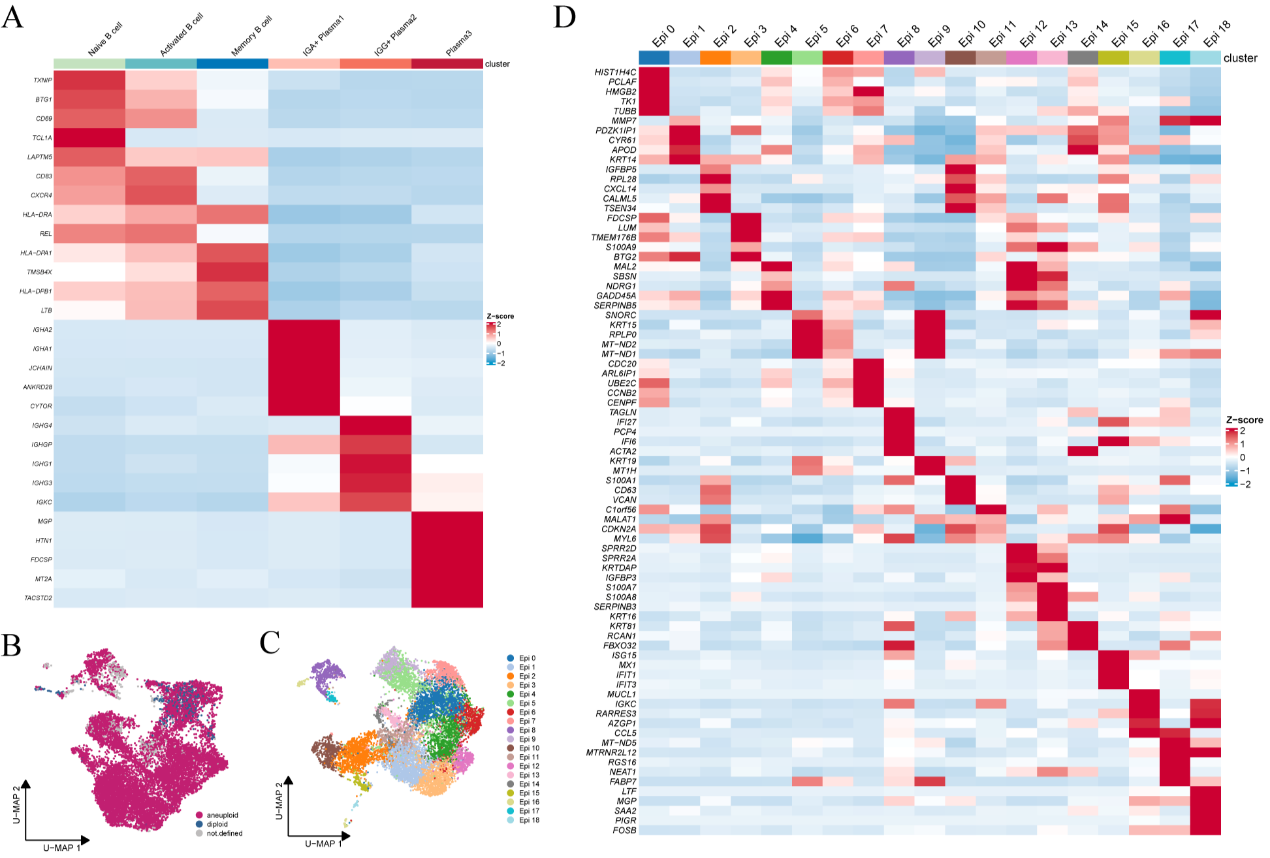


**Figure S6 Additional characteristics of B and epithelial cell clusters. (A)** Heatmap showing the top 10 differentially expressed genes for each B cell subpopulation. **(B)** Employing the CopyKAT algorithm to determine benign and malignant cells, shown by UMAP plot. **(C)** UMAP plot shows 19 cell clusters from malignant cells identified via CopyKAT, colored by cell cluster. **(D)** Heatmap showing the top 10 differentially expressed genes for each malignant cell subpopulation.


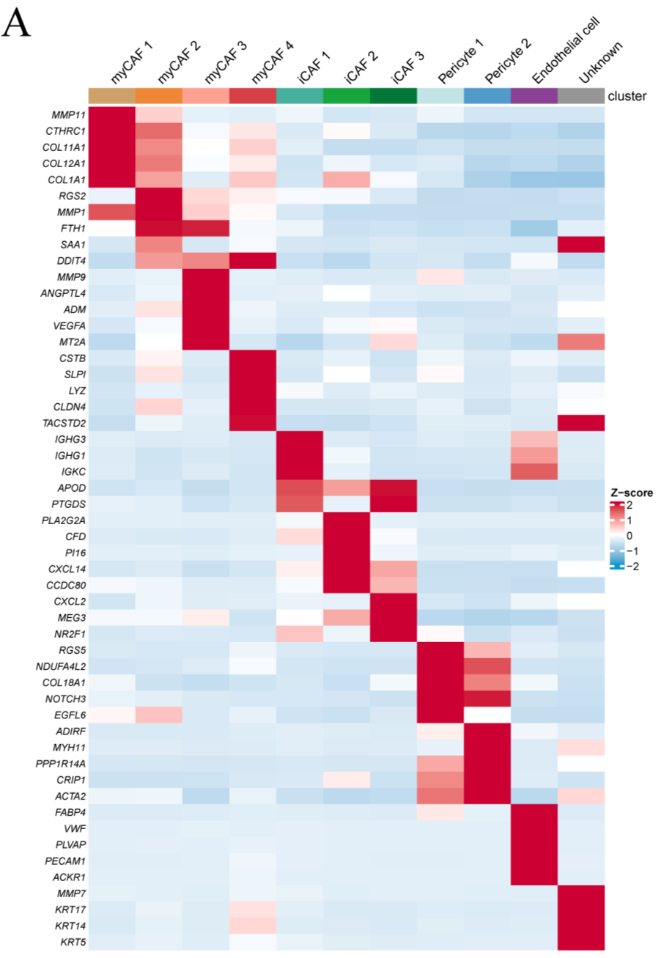


**Figure S7 Additional characteristics of stromal clusters.** Heatmap showing the top 10 differentially expressed genes for each stromal cell subpopulation.


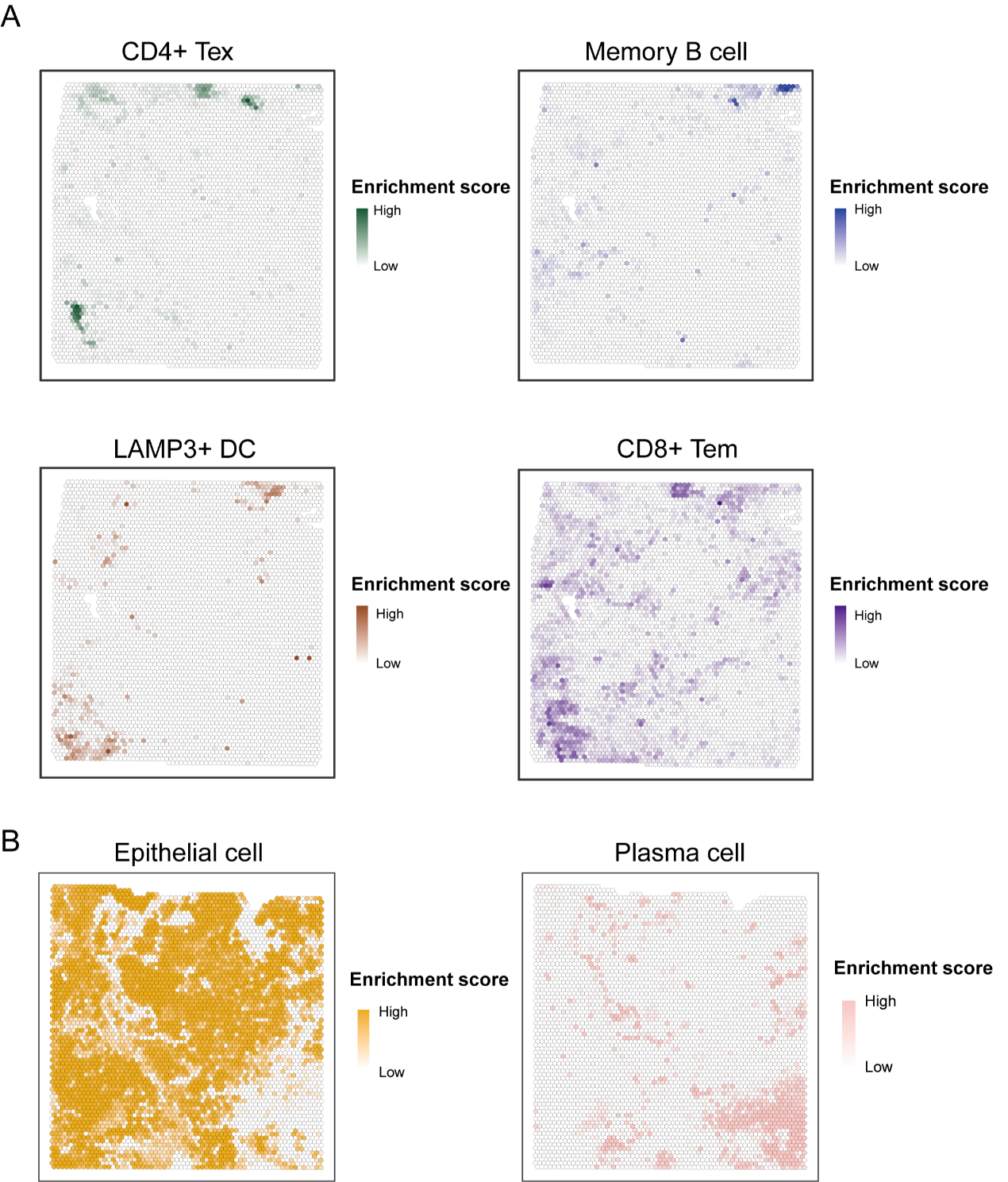


**Figure S8 Additional spatial correlation among cell types. (A)** Spatial distribution of enrichment scores for CD4+ Tex, Bm, LAMP3+ DC, and CD8+ Tem. **(B)** Spatial distribution of enrichment scores for epithelial cell and plasma cell.


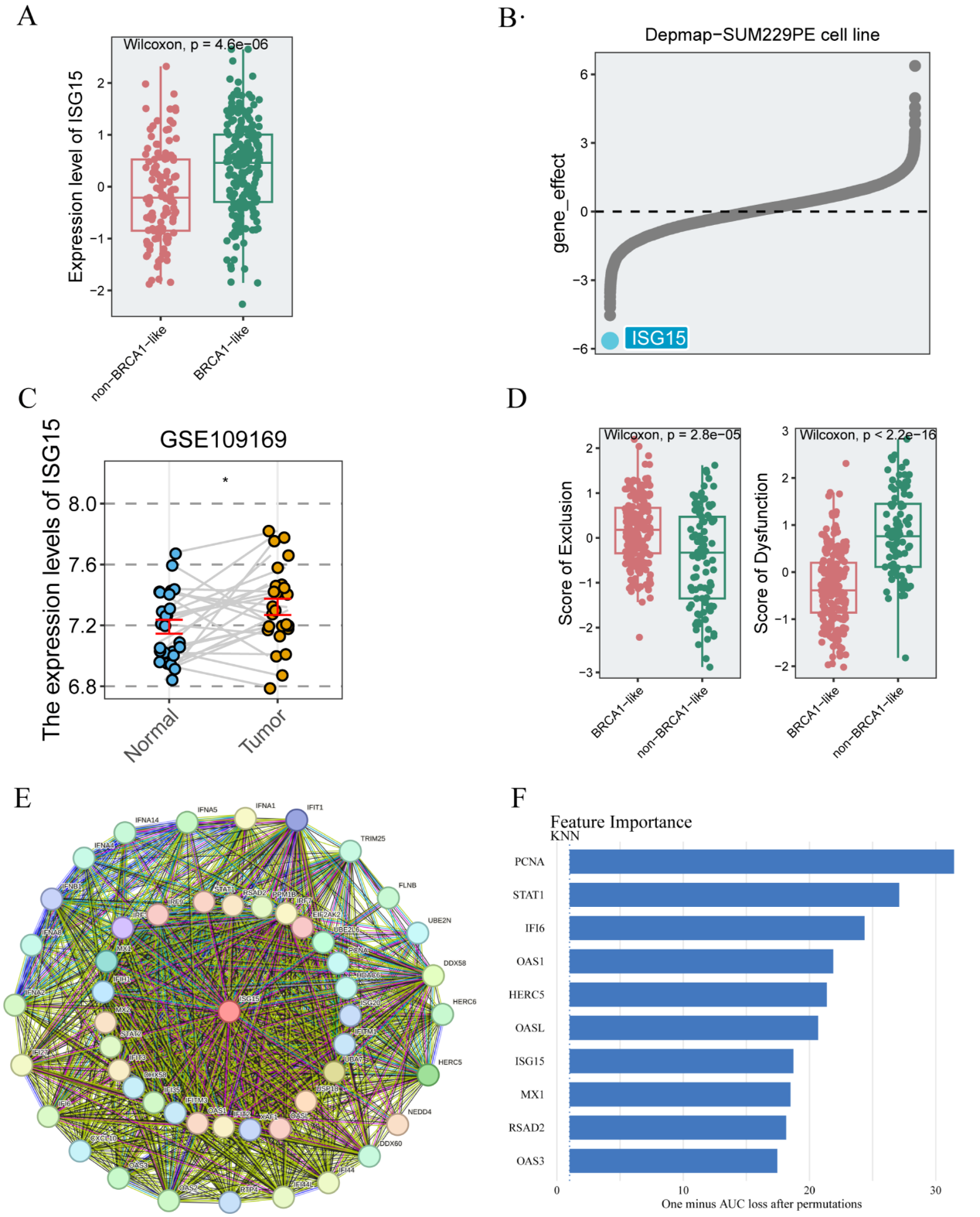


**Figure S9 The feature of ISG15 and predictive model. (A)** The difference of ISG15 expression between BRCA1-like group and non-BRCA1-like group in TNBC patients from METABRIC cohort. Wilcoxon rank-sum test. **(B)** The gene effect scores of SUM229PE cell line from DepMap database. Blue dot highlights the gene effect score of ISG15. The score is lower, the gene is more essential for the cell line. **(C)** Boxplot shows the paired differential expression of ISG15 between normal and tumor tissues from GSE109169. The grey line indicates the samples in two group is derived from one patient. T-test. **(D)** Boxplot depicts the difference of exclusion and dysfunction scores between BRCA1-like group and non-BRCA1-like group. Wilcoxon rank-sum test. **(E)** Protein–protein interactions network of ISG15-related genes constructed by the STRING database. **(F)** Feature importance scores of these genes in the predictive model.
